# Epigenetic and genetic factors modifying the association between diet quality and incident type 2 diabetes: the EPIC-Potsdam cohort

**DOI:** 10.1186/s12933-026-03356-0

**Published:** 2026-09-11

**Authors:** Christine El-Khoury, Fabian Eichelmann, Franziska Jannasch, Matthias B. Schulze

**Affiliations:** 1 Department of Molecular Epidemiology, German Institute of Human Nutrition Potsdam-Rehbruecke, Arthur-Scheunert-Allee 114-116, 14558 Nuthetal, Germany; 2 German Center for Diabetes Research (DZD), München-Neuherberg, Germany; 3 Institute of Nutritional Science, University of Potsdam, Nuthetal, Germany

**Keywords:** Diet quality, Type 2 diabetes, Polygenic risk score, Methylation risk score

## Abstract

**Background:**

Benefits of healthy diets for type 2 diabetes (T2D) prevention are established. It remains unclear whether such recommendations should be tailored to specific subgroups according to their susceptibility to the disease. This study aimed to assess whether the association of diet quality with T2D risk differs across subgroups with different genetic or epigenetic susceptibility.

**Methods:**

We used data from a case-cohort within the European Prospective Investigation into Cancer and Nutrition (EPIC)–Potsdam cohort (random subsample with genetic data of 2204 participants, 750 verified incident T2D cases; random subsample with epigenetic data of 1065 participants, 676 verified incident T2D cases). The Alternative Healthy Eating Index (AHEI-2010), Dietary Approaches to Stop Hypertension (DASH), Dietary Inflammatory Index (DII) and the Mediterranean Diet Pyramid score (MedPyr) were used to assess diet quality. A blood DNA methylation risk score (MRS) was used to reflect epigenetic risk. Genetic risk was characterized using global polygenic risk scores (PRS) and pathway-specific polygenic risk scores (pPRS). Cox proportional hazards models were used to assess the modification of the diet quality-diabetes risk association by the risk scores.

**Results:**

Higher adherence to the AHEI-2010 was associated with a lower risk of T2D, while higher MRS, PRS and pPRS predictably indicated higher risk in this population. We detected significant modification of the diet quality-T2D association by the MRS for AHEI-2010 (*p* = 0.008) and MedPyr (*p* < 0.0001) and DII (*p* = 0.010) but only the AHEI-2010 × MRS interaction was robust across all sensitivity analyses. However, we did not find evidence of interaction between diet quality and genetic risk.

**Conclusions:**

The associations of healthy diets with T2D could depend on the diabetes risk captured by blood DNA methylation profiles, but they do not seem to depend on the genetic predisposition for T2D. Confirmation of the effect modification by the MRS in independent populations is further required before considering clinical application.

**Supplementary Information:**

The online version contains supplementary material available at https://doi.org/10.1186/s12933-026-03356-0.

**Research insights**

**What is currently known about this topic?**

- While healthy diets are beneficial for T2D prevention, it is unclear whether dietary recommendations should be tailored according to individual susceptibility to T2D. Previous research has shown conflicting evidence on the interaction between diet and genetics on T2D, while evidence on the interaction between diet quality and epigenetics is lacking.

**What is the key research question?**

- Are the benefits of healthy diets on T2D modified by genetic or epigenetic factors?

**What is new?**

- We found modification of the diet quality-T2D association by a DNA methylation risk score. However, we did not find effect measure modification by the genetic risk for four dietary patterns.

**How might this study influence clinical practice?**

- Healthy diets seem to be beneficial for T2D regardless of the genetic predisposition, while benefits could depend on epigenetic risk profiles, which needs to be confirmed in independent populations to support clinical application.

## Background

Dietary prevention strategies to reduce type 2 diabetes (T2D) incidence rely on recommendations to improve dietary quality [1]. One common method to study diet quality is the “a priori” approach, where diet quality indices (DQIs) are pre-defined to reflect overall dietary patterns. These indices are compositions of different foods and nutrients, frequently selected based on their association with disease endpoints. Many DQIs are in line with food-based recommendations for the prevention of major cardiometabolic diseases, including T2D, thus, score the intake of fruits, vegetables, whole grains and fish positively, while limiting the consumption of red and processed meats and sugar sweetened beverages [2].

Evidence on the benefits of such diets on T2D incidence has been consistently highlighted [3]. However, it is unclear whether uniform diet quality recommendations suffice or personalized diet advice is needed. In fact, the precision nutrition framework considers how diet affects health considering interindividual response differences [4]. One aspect of heterogeneity is related to “gene-environment interactions”, assuming that genetic and environmental factors, both independent and interacting, contribute to the etiology of most diseases [5].

T2D arises from interactions between lifestyle exposures and interindividual biological susceptibility. DNA methylation (DNAm) captures exposure- and age-informed variation in gene regulation, and diet alters DNAm [6]. Specific DNAm profiles—summarized as methylation risk scores (MRS)—predict incident T2D [7–9]. Although higher diet quality is generally associated with lower T2D risk, emerging evidence suggests that the magnitude of this association may vary across individuals. DNAm is a biologically plausible mechanism for such heterogeneity because methylation changes have been linked to genes and pathways involved in insulin signaling, β-cell function, lipid metabolism, and inflammation, and diet-related exposures can influence methyl-donor availability and epigenetic regulation [10, 11]. Furthermore, previous studies indicate that dietary effects on cardiometabolic health might vary according to DNAm-associated traits [12–16]. Taken together, these findings support the hypothesis that DNAm patterns may serve as a biological proxy for the metabolic shifts that determine how diet quality influences the risk of incident T2D. To our knowledge, no previous study has assessed statistical interaction between diet and MRS on T2D risk.

Unlike DNAm profiles which are dynamic in response to environmental and lifestyle exposures including diet, the genetic profile is a stable predisposing factor for T2D. The genetic predisposition for T2D can be assessed by polygenic risk scores (PRS), which combine the totality effect of (common) genetic variants associated with the disease. Furthermore, to capture the underlying pathophysiological pathways, partitioned polygenic risk scores (pPRS) have been developed [17]. The majority of previous studies on the modification of the effect of diet quality by genetic risk were limited to total PRS and thereby did not address the potential of pathway-specific modification of dietary benefit [18–24]. One study found that a higher diet quality was associated with a lower risk of T2D regardless of the genetic predisposition to the disease estimated by PRS and pPRS [25]. However, more recent work based on larger set of genome-wide association studies (GWAS) resulted in increased power to detect additional pathway-specific clusters, that were not captured by that study [26]. Moreover, other approaches to derive the pPRS have recently emerged, which were not previously assessed in this context [27].

While genetic risk scores capture inherited T2D susceptibility through genetic variants, methylation risk scores can reveal how environmental and lifestyle factors modify the gene expression. Thus, genetic risk scores cannot explain the non-hereditary T2D susceptibility, but methylation risk scores could capture dynamic gene-environment interactions that influence T2D risk, beyond independent stable genetic factors. The metabolic processes underlying T2D etiology therefore can vary according to both genetic and epigenetic risk profiles, and the response to dietary intake, which is also affected by those metabolic processes as previously mentioned, might then be different across subgroups with different genetic or epigenetic profiles. Considering both of these factors could offer a complementary perspective on T2D etiology.

Therefore, we aimed to assess whether the association of diet quality with T2D risk differs across genetic or epigenetic risk strata, defined by MRS and PRS/pPRS, respectively.

## Methods

### Study design and population

The European Prospective Investigation into Cancer and Nutrition–Potsdam (EPIC–Potsdam) cohort included 27,548 participants (16,644 women and 10,904 men) aged between 35 and 65 years, recruited from the general population of Potsdam, Germany between 1994 and 1998. Informed consent was obtained from all participants, and the study was approved by the Ethical Committee of the State of Brandenburg, Germany [28]. The baseline assessment included anthropometrics and blood pressure measurements, blood sampling, collection of dietary intake data, lifestyle and sociodemographic characteristics, and information on prevalent diseases via questionnaires and personal interviews. Plasma levels of TG and HDL-cholesterol as well as red blood cell HbA1c have been measured at baseline as previously described [29]. Participants were then actively followed-up every 2 to 3 years by mailed questionnaires or telephone, when necessary. Response rates ranged from 90 to 96% per follow-up round.

For efficient molecular phenotyping, a case-cohort was constructed. From the 26,437 participants who provided blood samples, a subcohort of 2500 participants was randomly drawn as representative of the full cohort. In addition, all incident T2D cases (n = 820) that occurred in the full cohort until August 31, 2005 were included for the analysis. Follow-up was defined as the time between enrollment and study exit due to T2D diagnosis, death, dropout or final censoring date, whichever came first. After excluding participants with prevalent diabetes, self-reported diabetes or diabetes medication at baseline, insufficient blood sample or missing follow-up, implausible energy intake (defined as < 500 kcal/d or > 3500 kcal/d for women; < 800 kcal/d or > 4200 kcal/d for men) and missing genetic or DNAm data, the analytical sample which included participants with genetic data included 2204 members of the subcohort and 750 incident T2D cases, including 71 overlapping cases, while the analytical sample which included participants with DNAm data consisted of 1065 individuals in the subcohort, 676 incident T2D cases including 26 overlapping cases (Additional file 1: Supplementary Fig. 1).

### Ascertainment of type 2 diabetes

Incident diabetes cases were identified through self-reported diagnosis, T2D-related medication or dietary treatment, death certificates, tumor centers, physicians or clinics. Based on these sources, potential diabetes cases were identified and a standard inquiry form was then sent to the treating physician. Only physician-verified T2D cases (ICD-10 code E11) diagnosed after the baseline examination were considered to be confirmed incident cases.

### Diet quality assessment

The habitual dietary intake over the last 12 months was assessed at baseline with a 149-food item semi-quantitative food frequency questionnaire (FFQ) [30]. The reproducibility and validity of the FFQ have been previously reported [31]. Diet quality was assessed using 4 different DQIs, as explained below. Detailed compositions of the 4 indices are reported in Additional file 1: Supplementary Table 1.

#### Alternative healthy eating index-2010 (AHEI-2010)

The AHEI-2010, as originally developed, includes 11 foods and nutrients associated with chronic disease risk [32]. However, due to the lack of data on trans-fat in our population, and the imprecise estimates of sodium consumption from FFQ, we calculated a 9-item AHEI-2010. Each item was scored from 0 to 10 according to pre-defined cut-offs of intake, and the total score could range from 0 (non-adherence) to 90 (highest adherence).

#### Dietary approaches to stop hypertension (DASH)

The DASH score, as structured by Fung et al., reflects the level of adherence to the DASH diet [33]. Our score included 7 out of the original 8 components, sodium being excluded due to imprecise estimates. The components were scored according to sex-specific quintiles of intake: the lowest quintile received 1 point, while the highest quintile received 5 points. The sum of individual component scores then constituted the overall DASH score, which could range from 7 (minimal-adherence) to 35 (highest adherence).

#### Mediterranean diet pyramid (MedPyr)

The score used to assess the adherence to the Mediterranean diet was based on the Mediterranean pyramid recommendations, and calculated based on the algorithm developed by Tong et al. [34] and previously used in EPIC-Potsdam [35, 36]. This score includes 15 foods and food groups, scored according to low, moderate or high intake (Additional file 1: Supplementary Table 1), and the overall score can range from 0 (non-adherence) to 15 (highest adherence).

#### Dietary inflammatory index (DII)

The inflammatory potential of the diet was assessed via the DII, with 29 components available from the original 45 [37]. In brief, the centered percentile value for each food parameter was calculated based on dietary intake data and world average and standard deviation. Each parameter-specific value was then multiplied by its corresponding literature-derived inflammatory effect score to obtain the food parameter-specific scores, which were then summed up to calculate the overall DII score.

### Methylation risk score for type 2 diabetes

Details on DNAm analysis in our population have been previously reported [38]. Among the 145 CpG sites included in the original score, 144 were included in the MRS applied to our data, after QC filtering and exclusion of missing CpG sites. We calculated the MRS for T2D developed by Cheng et al. [39]. DNAm data generated with Illumina 850 k array were available for 1715 participants, including 676 incident T2D cases. Quality control and normalization were performed with *meffil* using default settings [40]. The score was calculated as a weighted sum of the available CpG sites, with the selected sites and respective weights reported in Additional file 2: Supplementary File 1. The MRS was then z-standardized (mean = 0, SD = 1).

### Polygenic risk scores for type 2 diabetes

Samples from EPIC-Potsdam participants have been genotyped, with details on DNA extraction, genotyping and quality control measures reported elsewhere [41]. We implemented the PGS pipeline *pgsc_calc* to calculate PRS and pPRS via custom scoring files [42]. In short, the pipeline handles harmonization of genotypes and scoring files including strand flip, SNP matching and scoring in a robust and reproducible manner. In order to generate the pPRS, we used SNP selection and respective weights from two previous studies which identified each a set of pPRS related to T2D underlying pathophysiological pathways. One set of 8 pPRS was generated by hard clustering, referred to as Suzuki-pPRS, where each SNP is allocated to one cluster [27]. The other set included 14 pPRS and was generated by a soft clustering approach, referred to as Smith-pPRS, where each SNP can be allocated to more than one cluster according to the probability of belonging to each cluster [26]. A list of the genetic variants used to construct each pPRS is available in Additional file 3: Supplementary File 2 and Additional file 4: Supplementary File 3. We applied a rank-based inverse normal transformation to the PRS and pPRS.

### Statistical analysis

Partial Spearman correlations, adjusted for age and sex, were used to assess pairwise associations between DQIs, PRS, and MRS. The prospective associations between the DQI scores, PRS/pPRS and MRS with incident T2D were assessed with multivariable Cox proportional hazards models. The DQI scores were modeled continuously (by 1-SD increment) and categorically (quintile comparison, with quintile 1 (Q1) as reference). Additionally, restricted cubic splines were used to detect non-linear associations of the DQIs with incident T2D. The likelihood ratio test was used to compare the fit between the linear and cubic spline models. A *p*-value < 0.05 indicated a non-linear association, which was then plotted.

The case-cohort design was accounted for using Prentice weighting. Age was the underlying time variable, entry time was age at baseline, and exit time was age at event or censoring. Potential ties in failure time were handled according to Efron approximation. The proportional hazards assumption was verified by graphical inspection of Schoenfeld residuals plots and was confirmed for all models. The models were fitted as follows; model 1 was adjusted for age and sex. Model 2 was additionally adjusted for education (no vocational training/vocational training, technical college, university), physical activity (sum of sports, biking and gardening in hours/week), smoking status (never smoker, former smoker, current smoker), energy intake (kcal/day) and alcohol consumption (non-drinker, low [≤ 6 g/day], moderately low [> 6 to ≤ 12/g/day], moderately high [> 12 to ≤ 24/g/day], high [> 24 to ≤ 60 g/day], very high [> 60 g/day]). Specifically, energy or alcohol were included in the adjustment set only when they were not index components. We considered adiposity to be on the mediating path between diet quality and incident T2D and, thus, did not adjust for adiposity measures in the main analyses. MRS analyses were additionally adjusted for blood cell proportions.

The interaction between the diet scores and the PRS/pPRS and MRS, was tested in multiplicative interaction analyses. For DQIs that showed linear association with T2D, the DQI was modeled as a continuous variable, and interaction was assessed by including a multiplicative interaction term (DQI × continuous PRS or MRS) in the fully adjusted model. For DQIs with evidence of a non-linear association with T2D, the DQI was modeled as a categorical variable (tertiles), and interaction was assessed by comparing the main Cox models with and without the DQI × PRS or MRS interaction terms using a likelihood ratio test. To account for multiple comparisons, false discovery rate (FDR) correction was applied separately by DQI, correcting across all PRS tested within each DQI.

In stratified analyses, we assessed the association of the 4 DQIs (high vs. low tertile) with incident T2D across PRS/pPRS and MRS categories, separately. These analyses were stratified by the corresponding score tertiles according to the subcohort distribution (for each PRS, pPRS and MRS).

In sensitivity analyses, we excluded participants with prevalent myocardial infarction or stroke at baseline to verify the consistency of results. Two additional sensitivity analyses were carried out for MRS; in one analysis, we excluded participants with high baseline HbA1c (≥ 6.5%) to minimize reverse causality. In the other analysis, an additional model was fitted as the main model and was further adjusted for BMI (continuous, kg/m^2^), HbA1c (continuous log-transformed, %), TG and HDL-cholesterol (both continuous log-transformed, mg/dL).

All statistical analyses and plots were done using R software, version 4.3.0.

## Results

### Descriptive characteristics

The subcohort consisted of 2204 individuals with complete genetic data (62% women) with a median age of 49.6 (IQR 42.1–57.8). The corresponding baseline characteristics are summarized for the subcohort and incident T2D cases in Additional file 1: Supplementary Table 2, and across extreme quintiles (Q1 vs. Q5) of the four assessed dietary pattern scores in Table 1. For most DQIs, both Q1 and Q5 included a higher proportion of women, with the exception of DII. Participants in the Q5 of DASH and DII were older than those in Q1, while the opposite was found for AHEI-2010 and MedPyr. Most participants in Q5 of the healthy DQIs had a university degree and were more active. Across all 4 DQIs, participants in the highest quintile had a lower waist circumference compared to those in the lowest quintile, and the majority of participants in both Q1 and Q5 were never smokers. The descriptive characteristics for the analytical sample with complete DNAm data were quite comparable and are shown in Additional file 1: Supplementary Table 3.

**Table 1:** Baseline subcohort characteristics across extreme quintiles of the assessed dietary patterns

Characteristics	DASH		DII		AHEI 2010		MedPyr	
	Q1 (N = 441)*	Q5 (N = 440)*	Q1 (N = 441)*	Q5 (N = 440)*	Q1 (N = 441)*	Q5 (N = 440)*	Q1 (N = 441)*	Q5 (N = 440)*
Sex								
Male	176 (40%)	144 (33%)	239 (54%)	90 (20%)	181 (41%)	144 (33%)	178 (40%)	157 (36%)
Female	265 (60%)	296 (67%)	202 (46%)	350 (80%)	260 (59%)	296 (67%)	263 (60%)	283 (64%)
Age, years	47.2 (40.8, 56.2)	52.9 (43.8, 59.5)	49.1 (42.7, 57.1)	49.5 (41.4, 57.3)	50.4 (42.0, 58.4)	50.1 (42.9, 57.9)	51.9 (42.7, 59.1)	47.9 (42.3, 56.2)
Waist circumference, cm	85.0 (74.5, 94.0)	84.0 (75.0, 93.0)	88.0 (77.5, 95.0)	82.0 (74.0, 91.0)	86.0 (76.0, 95.5)	84.0 (74.0, 93.0)	86.0 (76.0, 96.0)	83.8 (74.0, 92.3)
BMI, kg/m^2^	25.3 (22.7, 28.2)	25.6 (23.2, 28.0)	25.6 (23.4, 28.2)	25.3 (22.9, 28.8)	25.9 (23.1, 28.9)	25.4 (22.9, 28.1)	25.7 (22.9, 28.7)	25.2 (23.1, 28.0)
Smoking status								
Never-smoker	164 (37%)	244 (55%)	204 (46%)	206 (47%)	182 (41%)	224 (51%)	204 (46%)	229 (52%)
Current sSmoker	138 (31%)	62 (14%)	84 (19%)	113 (26%)	137 (31%)	66 (15%)	99 (22%)	67 (15%)
Former smoker	139 (32%)	134 (30%)	153 (35%)	121 (28%)	122 (28%)	150 (34%)	138 (31%)	144 (33%)
Educational status								
Technical college	112 (25%)	133 (30%)	99 (22%)	110 (25%)	115 (26%)	118 (27%)	103 (23%)	112 (25%)
University	146 (33%)	169 (38%)	193 (44%)	132 (30%)	124 (28%)	194 (44%)	128 (29%)	202 (46%)
No/vocational training	183 (41%)	138 (31%)	149 (34%)	198 (45%)	202 (46%)	128 (29%)	210 (48%)	126 (29%)
Physical activity, h/wk	3.5 (1.0, 6.5)	5.5 (2.8, 9.0)	5.5 (3.0, 9.0)	3.5 (1.0, 6.5)	4.0 (1.5, 7.5)	5.5 (3.0, 9.0)	4.5 (2.0, 8.0)	5.0 (3.0, 8.5)
Prevalent hypertension	193 (44%)	222 (50%)	222 (50%)	192 (44%)	222 (50%)	204 (46%)	222 (50%)	204 (46%)
Prevalent dyslipidemia	105 (24%)	131 (30%)	115 (26%)	112 (25%)	116 (26%)	122 (28%)	142 (32%)	106 (24%)
Alcohol intake, g/day	8.6 (3.1, 21.3)	6.3 (2.2, 15.3)	13.5 (5.2, 25.1)	4.6 (1.5, 10.1)	3.1 (0.9, 25.2)	9.7 (5.8, 16.5)	5.2 (1.6, 22.9)	10.1 (4.5, 18.7)
Total energy intake, kcal/day	1987 (1632, 2471)	2032 (1730, 2490)	2676 (2296, 3107)	1457 (1264, 1671)	2055 (1678, 2504)	1932 (1612, 2358)	1959 (1539, 2456)	2118 (1752, 2565)
DASH	15.0 (2.0)	27.5 (1.9)	23.9 (4.3)	18.8 (3.8)	18.5 (4.2)	24.5 (3.7)	19.3 (4.2)	23.7 (4.2)
DII	1.2 (1.6)	-0.8 (1.3)	-2.0 (0.6)	2.7 (0.7)	1.0 (1.6)	-0.4 (1.6)	0.9 (1.7)	-0.6 (1.5)
AHEI 2010	31.0 (6.8)	42.9 (8.2)	40.7 (8.4)	33.5 (8.6)	24.9 (3.7)	49.4 (3.7)	32.2 (8.1)	42.7 (7.9)
MedPyr	6.5 (1.1)	7.6 (1.0)	7.5 (1.1)	6.5 (1.0)	6.4 (1.0)	7.7 (1.0)	5.5 (0.6)	8.6 (0.5)

*n (%); Median (Q1, Q3); Mean (SD). DASH: Dietary Approaches to Stop Hypertension; DII: Dietary Inflammatory Index; AHEI: Alternative Healthy Eating Index; MedPyr: Mediterranean Diet Pyramid

The mean (± SD) diet scores across the subcohort were 21.3 (± 4.4) for DASH, 37 (± 8.7) for AHEI-2010, 0.3 (± 1.7) for DII and 7 (± 1.1) for MedPyr (Additional file 1: Supplementary Table 2). Moderate correlations were found between the 4 DQIs, where DII was inversely correlated with the other diet scores, and pairwise partial spearman correlation coefficients ranged from − 0.47 between DII and DASH, to 0.49 between AHEI-2010 and DASH (Additional file 1: Supplementary Fig. 2).

The two global PRS were strongly correlated (r = 0.67), while correlations between the different pPRS ranged from − 0.08 to 0.62 (Additional file 1: Supplementary Fig. 2). Weak correlations were found between the MRS and the global PRS (r = 0.09 and r = 0.07 with Suzuki-PRS and Smith-PRS, respectively), and with each of the pPRS. In addition, correlations between the assessed DQIs and each of the polygenic and epigenetic risk scores were weak (Additional file 1: Supplementary Fig. 2).

### Diet quality and type 2 diabetes risk

The association between DII and T2D risk was non-linear, thus modelled as restricted cubic spline (Additional file 1: Supplementary Fig. 3). As for the remaining 3 DQIs, which showed linear associations with T2D, they were modelled continuously per 1-SD increment. No statistically significant associations were found between the four DQIs, modeled continuously per 1-SD increment, and diabetes risk in the fully adjusted model. When the DQIs were modelled as categorical variables using quintile contrast, a significant inverse association between AHEI-2010 and incident T2D was found in the fully adjusted model 2 (HR_Q5 vs. Q1_ 0.73, 95% CI 0.54–0.98) (Additional file 1: Supplementary Table 4). Excluding participants with baseline myocardial infarction or stroke in the main analyses showed similar results (Additional file 1: Supplementary Table 5).

### Genetic and epigenetic scores and type 2 diabetes risk

The MRS was associated with higher risk of T2D, both in the age, sex and blood cell proportions-adjusted model (HR_per 1 SD_ 2.45 (2.16–2.78)) and in the main multivariable-adjusted model (HR_per 1 SD_ 2.69 (2.37–3.05)). Additionally adjusting for BMI, HbA1c, TG and HDL-cholesterol attenuated the association to 1.58 (1.34–1.85). The HR per 1 SD (95% CI) was 2.40 (2.08–2.76) after excluding participants with baseline HbA1c ≥ 6.5%, and 2.70 (2.37–3.07) after excluding those with prevalent CVD. The global PRS were associated with higher risk of T2D with an age- and sex- adjusted HR_per 1 SD_ of 1.84 (1.67–2.03) for the Suzuki-PRS and 1.70 (1.55–1.87) for the Smith-PRS. A higher genetic risk characterized by the pPRS was generally associated with a higher risk of T2D; this was consistent across all the Suzuki-pPRS, with HR_per 1 SD_ ranging from 1.14 (1.05–1.25) for the “liver and liver metabolism” score, to 1.35 (1.23–1.48) for the “betacell + proinsulin pathway” score. Most Smith-pPRS were significantly associated with a higher T2D risk ((HR_per 1 SD_ ranging from 1.10 (1.01–1.21) for the “cholesterol” score to 1.33 (1.22–1.45) for the “proinsulin” and 1.33 (1.22–1.46) for the “beta cell 2” scores), with the exception of the “ALP negative”, “bilirubin” and “liver-lipid” scores (Additional file 1: Supplementary Fig. 4). Similar results were found after exclusion of prevalent CVD cases at baseline (Additional file 1: Supplementary Fig. 5).

### Interaction between diet quality and methylation risk score

**Fig. 1:**
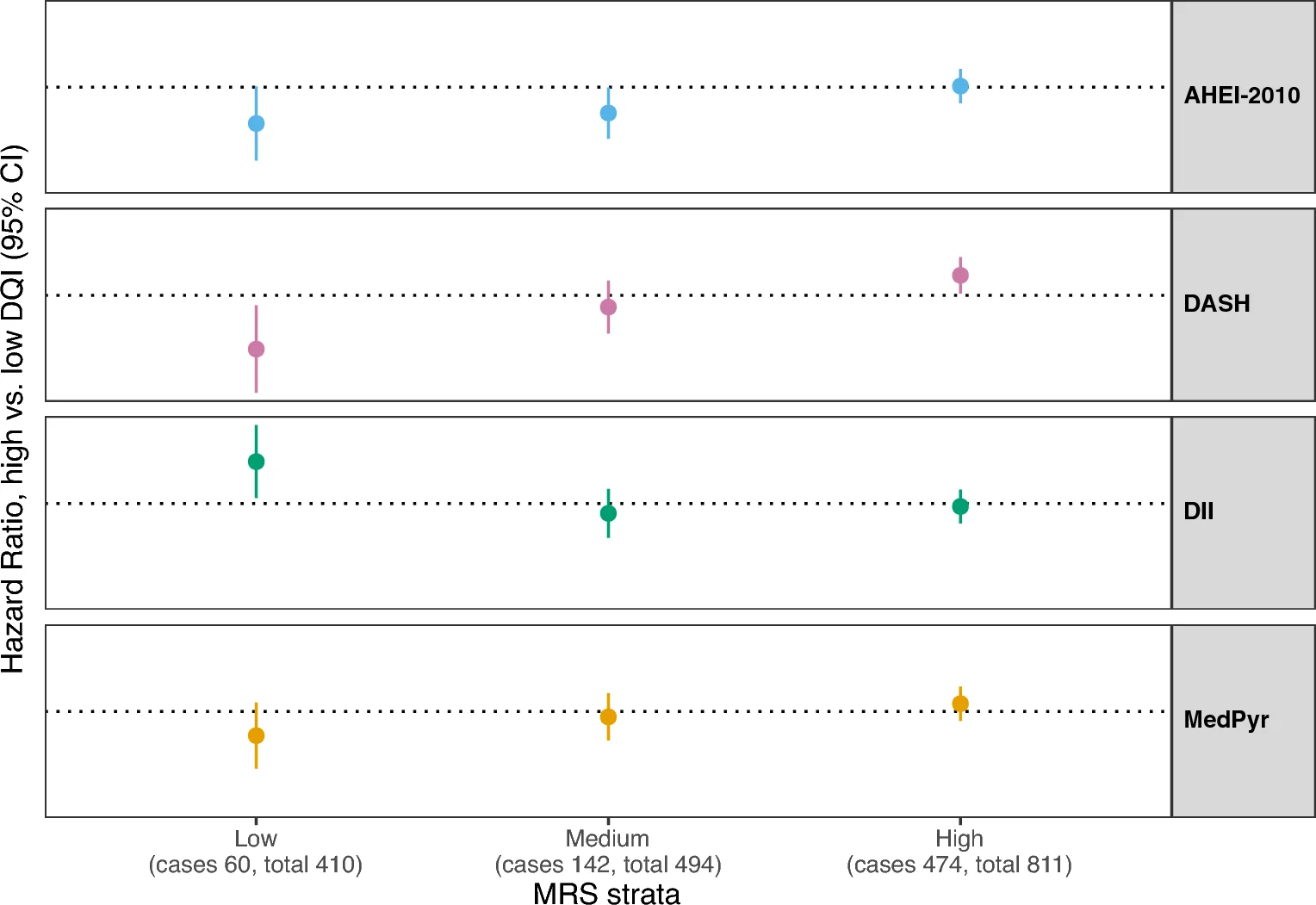
Association between the assessed DQIs and incident type 2 diabetes across tertiles of methylation risk score. Model adjusted for age (as underlying time variable), sex, proportions of B lymphocytes, CD4+ T lymphocytes, CD8+ T lymphocytes, monocytes, and natural killer (NK) cells, education, physical activity, smoking status, energy intake (except DII), alcohol intake (DASH only). DASH: Dietary Approaches to Stop Hypertension; DII: Dietary Inflammatory Index; AHEI: Alternative Healthy Eating Index; MedPyr: Mediterranean Diet Pyramid. MRS: methylation risk score

For DQIs that showed linear association with T2D, significant interaction was found between the MRS and AHEI-2010 (*p* = 0.008) and MedPyr (*p* < 0.0001), but not with DASH (*p* = 0.348). For the DII, which was non linearly associated with T2D, there was a significant interaction with MRS (*p* = 0.010)). Analyses stratified by tertiles of MRS based on the main adjustment model showed that a higher adherence to AHEI-2010 tended to associated with a lower risk of T2D in participants with low and medium MRS, but these associations did not reach statistical significance, and no clear pattern was observed for the MedPyr or DII (Fig. 1 and Additional file 1: Supplementary Table 6). In sensitivity analyses, many of the interactions remained consistent, however, additionally adjusting for BMI, HbA1c, TG and HDL-cholesterol impacted most interactions, with the exception of AHEI-2010 × MRS, which was robust across all models (Additional file 1: Supplementary Table 7).

### Interaction between diet quality and polygenic risk scores

In the main analyses, there was no evidence of significant interaction between diet quality and T2D genetic risk (Additional file 1: Supplementary Tables 8 and 9). Similarly, none of the interaction terms were significant in multiplicative interaction analyses based on the main model in the case-cohort excluding prevalent CVD cases at baseline (Additional file 1: Supplementary Table 10).

**Fig. 2:**
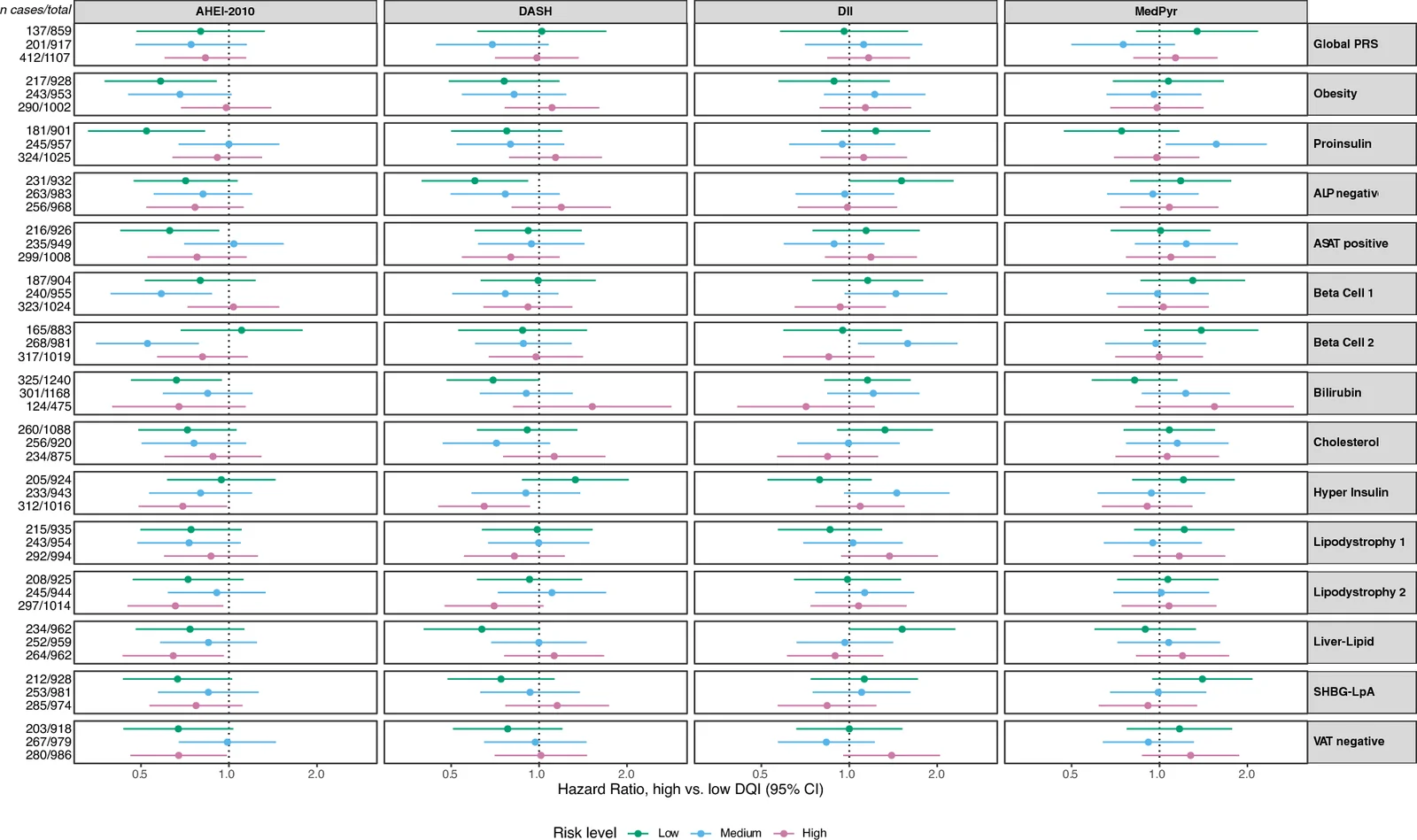
Association between the assessed DQIs and incident type 2 diabetes across tertiles of genetic risk defined by soft clustering-derived scores. Model adjusted for age (as underlying time variable), sex, education, physical activity, smoking status, energy intake (except DII), alcohol intake (DASH only). DASH: Dietary Approaches to Stop Hypertension; DII: Dietary Inflammatory Index; AHEI: Alternative Healthy Eating Index; MedPyr: Mediterranean Diet Pyramid

**Fig. 3:**
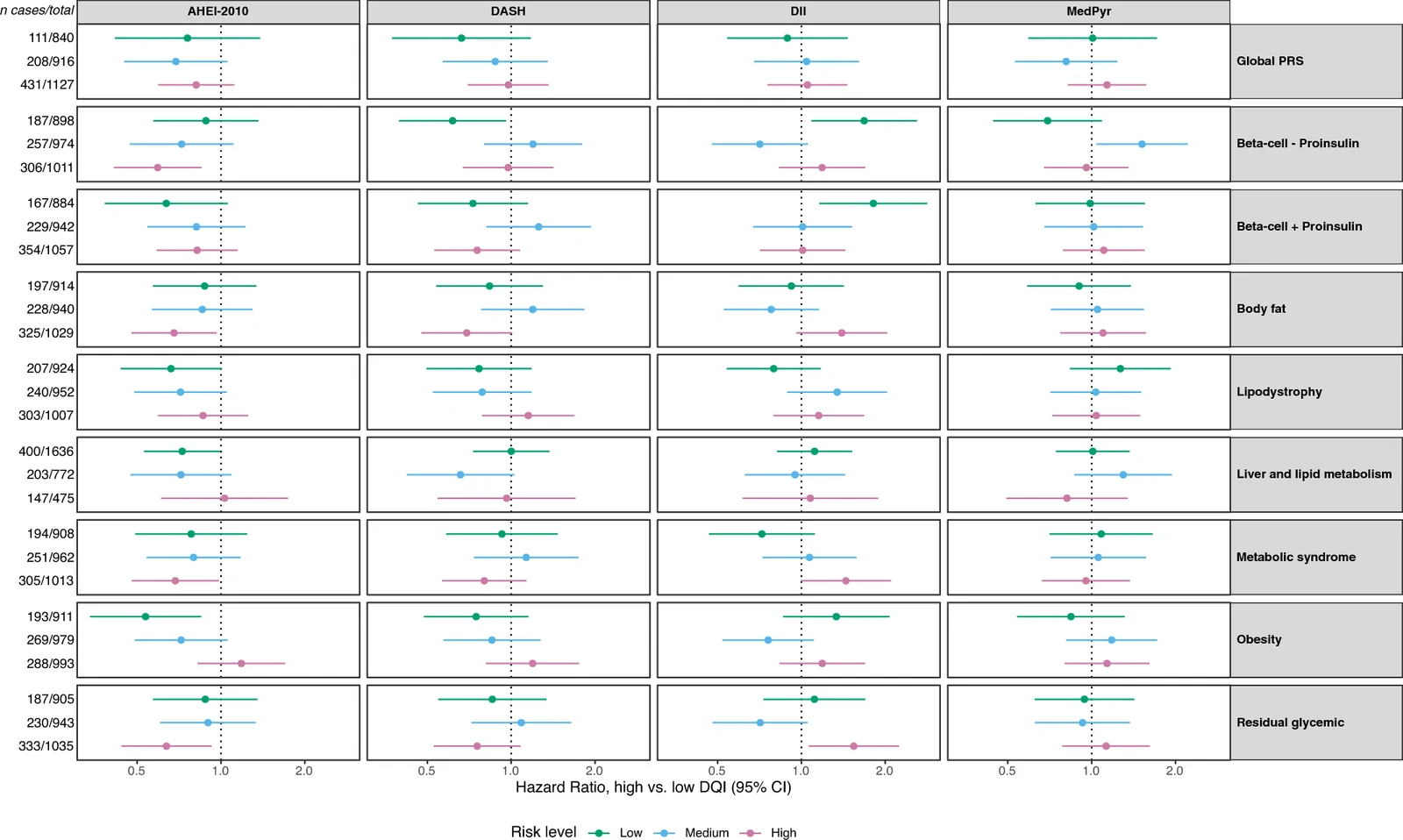
Association between the assessed DQIs and incident type 2 diabetes across tertiles of genetic risk defined by hard clustering-derived scores. Model adjusted for age (as underlying time variable), sex, education, physical activity, smoking status, energy intake (except DII), alcohol intake (DASH only). DASH: Dietary Approaches to Stop Hypertension; DII: Dietary Inflammatory Index; AHEI: Alternative Healthy Eating Index; MedPyr: Mediterranean Diet Pyramid

Subsequent analyses based on the main adjustment model and stratified by genetic risk, defined by each genetic risk score separately, generally showed similar direction of associations of the assessed DQIs across most genetic risk subgroups defined by the pPRS. Similarly, no distinct pattern was found across subgroups of genetic risk defined by the global PRS. Overall, the healthy dietary patterns, namely AHEI-2010, DASH and MedPyr tended to show inverse associations with T2D regardless of the genetic risk, while the DII tended to be positively associated with T2D risk (Figs. 2 and 3, Additional file 1: Supplementary Tables 8 and 9).

## Discussion

In this study, we found evidence of interaction between the MRS and diet quality assessed by AHEI-2010, MedPyr and DII on T2D risk, while only the AHEI-2010 × MRS interaction was robust across all sensitivity analyses. In particular, the AHEI-2010 association with T2D tended to be more pronounced among those in the low and medium MRS subgroups in the main analysis. In contrast, we did not find evidence of interaction between diet quality and genetic risk on the development of T2D.

Evidence on healthy dietary patterns for the prevention of T2D is well established, and supported by our results, particularly the AHEI. Such diets are characterized by higher intake of fruits, vegetables, whole grains and fish, and a limited consumption of red and processed meats, and sugar-sweetened beverages [2]. This dietary composition seems to improve insulin sensitivity, glucose metabolism, modulate gut microbiota and limit inflammation and oxidative stress [43].

Our findings suggest that the diet associations could vary according to individuals’ DNAm risk profiles. In particular, our stratified analyses showed that the benefits of diet quality, as assessed by AHEI-2010, appeared to be limited to participants with a low-medium methylation risk score, however the associations did not reach statistical significance. Nevertheless, due to the limitations of categorizing continuous exposures and the relatively smaller sample size in the strata, the direction of the associations across the different MRS strata could not be clearly established. DNAm profiles integrate accumulated metabolic burden from prior diet quality, obesity status, physical inactivity, as well as aging and intrauterine environment [6], and could serve as a biomarker of past exposure [44]. In this sense, differential methylation at loci regulating key metabolic pathways, including inflammation, adiposity and other metabolic processes linked to T2D could reflect cumulative effects of past exposures on metabolism and cardiometabolic health. Epigenetic dysregulation can induce transcriptional alterations that disrupt metabolic homeostasis in key tissues, including insulin-producing pancreatic beta-cells, and insulin-sensitive tissues such as liver, skeletal muscle and adipose tissue. This may lead to impaired insulin secretion, insulin resistance, reduced glucose uptake in skeletal muscle, increased glucose output in the liver and impaired glucose and lipid metabolism [10, 45]. Therefore, the epigenetic alterations and their associated metabolic phenotypes could disrupt the responses to diet and the physiological adaptation to nutrient intake. Consequently, such alterations may contribute to the interindividual variability in responses to diet and long-term T2D risk.

A key strength of the MRS is that it integrates signals from multiple past exposures, from in utero throughout adulthood, into a single composite measure, capturing cumulative metabolic and lifestyle burden that individual conventional biomarkers, measured at a single time point, cannot fully reflect. Because the MRS incorporates signals related to HbA1c, BMI, HDL-cholesterol, and TG, which themselves reflect accumulated metabolic burden, we conducted two additional analyses to verify the robustness of the results to metabolic confounding and reduce the possibility of reverse causation due to subclinical metabolic dysfunction. Additionally adjusting the main model for HbA1c, BMI, HDL-cholesterol, and TG attenuated but did not eliminate the MRS–T2D association, which remained statistically significant. Excluding individuals with baseline HbA1c ≥ 6.5% yielded similar results. In both analyses, MRS × AHEI-2010 interaction remained significant. While this supports the robustness of the findings, it does not fully establish the incremental clinical value nor the biological independence of the MRS, neither does it eliminate the possibility of reverse causation, which should be taken into consideration when interpreting the results.

Our findings suggest that the association of healthy diet with T2D may be independent of the genetic risk of T2D, which is consistent with several previous studies that used global PRS [19–21, 24, 25]. In contrast, one UK Biobank study on 357,419 individuals found evidence of interaction between a 10 component- diet quality score and a global PRS, with beneficial effects on diabetes risk evident in participants with a higher genetic risk [22]. Another UK Biobank study, including 126,203 individuals with normoglycemia and 16,068 with prediabetes, found evidence of interaction between a reduced rank regression-derived inflammatory diet and a genetic risk score in the normoglycemic subgroup only [23]. A study including 1196 T2D cases and 1337 matched controls in the Health Professionals Follow-Up Study found significant interaction between a Western dietary pattern and a genetic risk score including 10 SNPs, while no interaction was found with a prudent diet [18]. To our knowledge, only one previous study has investigated interactions with five pPRS which overlap with some of the Smith-pPRS used in our study; among 35,759 adults in 3 US cohorts, no evidence of additive or multiplicative interaction with AHEI or DASH on T2D risk was found [25]. In our study, stratified analysis showed similar direction of associations across risk strata, which supports the benefits of healthy diets on T2D regardless of the genetic susceptibility.

This study has several strengths. We investigated statistical interaction between several DQIs and MRS on the one hand, and new-generation PRS on the other hand, allowing not only to quantify the global genetic risk of T2D, but also the risk related to specific pathophysiological pathways involved in T2D, thereby reflecting its etiological heterogeneity. This combination might help to reveal the heterogeneous mechanisms behind T2D risk by capturing genetic susceptibility, gene-environment interactions and how such factors might affect the response to diet. In particular, the MRS integrates information from several DNAm sites into a single measure, providing a more comprehensive assessment of cumulative long-term biological changes than individual metabolic markers [46]. We also evaluated pPRS derived from two clustering approaches, which were mostly low to moderately correlated, capturing the complex pathophysiology of T2D. Furthermore, by combining PRS, pPRS and MRS, our multifaceted analysis in a prospective case-cohort design offers a more comprehensive understanding of T2D etiology.

We acknowledge some limitations. First, since this study is observational, residual confounding might be present. Second, dietary data was collected at baseline, which might not reflect dietary intake during the follow-up well. Third, several diet associations were in the expected direction, but did not reach statistical significance. This might reflect lower statistical power due to the set-up of the case-cohort compared to previous analyses on DQI in this cohort [35]. Fourth, the AHEI-2010, DII and DASH were modified compared to their original versions since some components were not available in our data. This could limit comparability with other studies that use different index compositions, however, most core aspects of the diets were preserved and most T2D-related components were retained. Noteworthy in this context, heterogeneity of DQI composition did not substantially influence the direction or strength of disease associations in prospective studies [3]. Fifth, our population is of European ancestry, so the observed associations might not generalize to populations of different ancestry.

## Conclusion

In conclusion, we found evidence that the association of healthy diets with T2D could depend on the disease risk captured by blood DNAm profiles, while this association did not seem to depend on the genetic predisposition for T2D. However, analyses stratified by MRS tertiles did not identify a statistically clear subgroup-specific dietary benefit. Therefore, these findings should be considered as hypothesis-generating, and further studies in independent populations with larger samples are needed, to support the clinical benefit of considering epigenetic profiles in tailoring dietary recommendations for T2D prevention.

## Supplementary Information

**Additional file 1: Supplementary Material.** El-Khoury_Supplementary_Material.pdf, supplementary tables and figures.

**Additional file 2: Supplementary File 2.** El-Khoury_S.File1_MRS_weights.csv, CpG sites and respective weights.

**Additional file 3: Supplementary File 3.** El-Khoury_S.File2_suzuki_log.csv, list of the genetic variants used to construct each hard clustering-derived pPRS.

**Additional file 4: Supplementary File 4.** El-Khoury_S.File3_smith_log.csv, list of the genetic variants used to construct each soft clustering-derived pPR.

## Data Availability

The data analyzed in the current study is not publicly available due to data protection regulations. In accordance with German federal and state data protection regulations, analyses of EPIC-Potsdam data may be initiated upon an informal inquiry addressed to the secretariat of the Human Study Center (office.hsz@dife.de). Each request will then have to pass a formal process of application and review.

## Abbreviations

T2D: Type 2 Diabetes
DQI: Dietary Quality Index
DNAm: DNA methylation
MRS: Methylation Risk Score
PRS: Polygenic Risk Score
pPRS: Pathway-specific Polygenic Risk Score
GWAS: Genome-Wide Association Studies
EPIC: European Prospective Investigation into Cancer and Nutrition
FFQ: Food Frequency Questionnaire
AHEI: Alternative Healthy Eating Index
DASH: Dietary Approaches to Stop Hypertension
MedPyr: Mediterranean diet Pyramid
DII: Dietary Inflammatory Index
FDR: False Discovery Rate
