# Supplementary material for: Epigenetic and genetic factors modifying the association between diet quality and incident type 2 diabetes: the EPIC-Potsdam cohort
Source: Cardiovasc Diabetol. 2026 Sep 11;25:275. doi: 10.1186/s12933-026-03356-0 (PMC13563862; doi:10.1186/s12933-026-03356-0)
Supplement: Supplementary file 1 [file 12933_2026_3356_MOESM1_ESM.pdf]

Epigenetic and Genetic Factors Modifying the Association between Diet Quality and Incident Type 2 Diabetes: The EPIC-Potsdam Cohort – El-Khoury et al.

Supplementary Material

Contents

S. Figure 1: Case-cohort design flowchart 2
S. Figure 2: Partial correlations between the assessed DQIs, PRS/pPS and MRS 3
S. Figure 3: Restricted cubic spline for the association between DII and type 2 diabetes 4
S. Figure 4: Association between the polygenic risk scores (per 1 SD increment) and incident type 2 diabetes 5
S. Figure 5: Association between the polygenic risk scores (per 1 SD increment) and incident type 2 diabetes after excluding prevalent CVD cases 6
S. Table 1: Composition of the assessed DQIs 7
S. Table 2: Baseline characteristics of the analytical sample for the PRS analyses 10
S. Table 3: Baseline characteristics of the analytical sample for MRS analyses 11
S. Table 4: Association between the DQIs and incident type 2 diabetes, modelled continuously and per extreme quintile contrast 12
S. Table 5: Association between the DQIs and incident type 2 diabetes, modelled continuously and per extreme quintile contrast after excluding prevalent CVD cases 13
S. Table 6: Association between the assessed DQIs and incident type 2 diabetes across tertiles of MRS 14
S. Table 7: P-interaction values for DQIxMRS in main and sensitivity analyses 15
S. Table 8: Association between the assessed DQIs and incident type 2 diabetes across tertiles of genetic risk defined by soft clustering-derived scores 16
S. Table 9: Association between the assessed DQIs and incident type 2 diabetes across tertiles of genetic risk defined by hard clustering-derived scores 20
S. Table 10: P-interaction for DQIxPRS after excluding baseline CVD cases 23

S. Figure 1: Case-cohort design flowchart

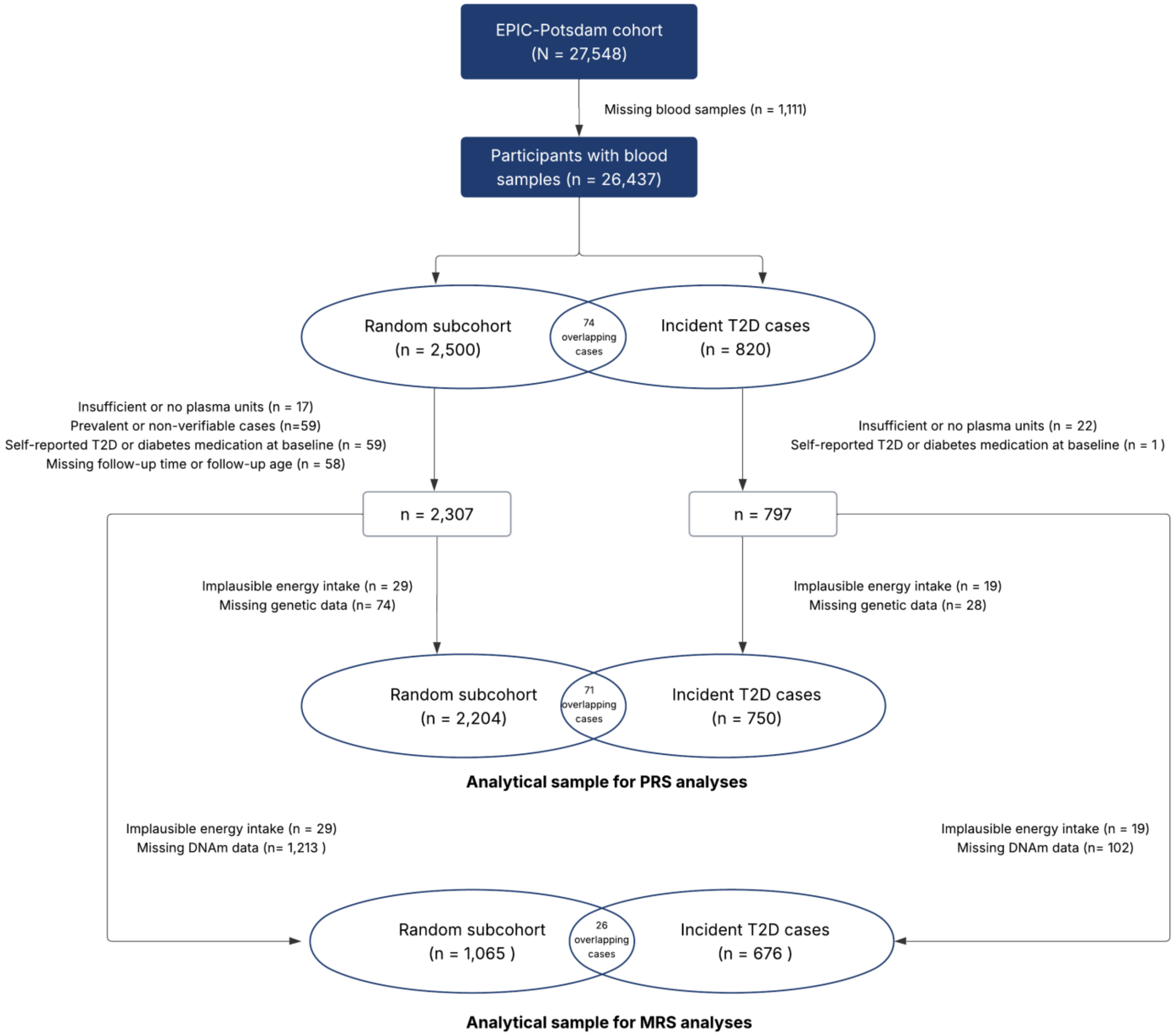

S. Figure 2: Partial correlations between the assessed DQIs, PRS/pPS and MRS

Correlation Matrix

Spearman r: partial (adjusted for age, sex)

	Suzuki Global PRS	Suzuki Beta-cell - Proinsulin	Suzuki Beta-cell + Proinsulin	Suzuki Body fat	Suzuki Lipodystrophy	Suzuki Liver and lipid metabolism	Suzuki Metabolic syndrome	Suzuki Obesity	Suzuki Residual glycemic	Smith Proinsulin	Smith Global PRS	Smith ALP negative	Smith ASAT positive	Smith Beta Cell 1	Smith Beta Cell 2	Smith Bilirubin	Smith Cholesterol	Smith Hyper Insulin	Smith Lipodystrophy 1	Smith Lipodystrophy 2	Smith Liver-Lipid	Smith Obesity	Smith SHBG-LpA	Smith VAT negative	MRS	DASH	DII	MedPyr	AHEI-2010
**AHEI-2010**	0.02	0.02	0.03	0.03	0.01	−0.01	−0.02	−0.02	0.01	−0.05	0.01	−0.01	−0.04	−0.04	0.05	0.00	−0.01	0.00	0.00	0.02	0.00	0.00	−0.02	0.01	−0.08	0.49	−0.33	0.39	1.00
**DASH**	−0.01	−0.02	−0.01	0.00	0.00	0.01	−0.02	0.03	0.02	−0.04	0.00	−0.04	0.02	−0.01	−0.01	−0.05	−0.01	0.03	−0.03	0.02	−0.05	0.05	0.02	0.01	−0.10	1.00	−0.47	0.33	0.49
**DII**	−0.02	0.02	−0.04	−0.04	0.02	0.04	0.00	0.02	−0.02	0.00	−0.02	0.06	−0.01	0.00	−0.05	0.03	0.06	−0.02	0.02	−0.02	0.06	−0.02	0.01	−0.04	0.03	−0.47	1.00	−0.34	−0.33
**MedPyr**	0.05	0.02	0.01	0.02	0.08	0.05	0.02	0.00	0.01	−0.03	0.03	0.00	0.01	−0.06	−0.01	−0.02	0.01	0.05	0.01	0.06	−0.04	0.04	−0.02	−0.02	−0.03	0.33	−0.34	1.00	0.39
**Suzuki Global PRS**	1.00	0.27	0.45	0.38	0.24	0.07	0.41	0.36	0.40	0.32	0.67	0.08	0.20	0.33	0.39	0.02	0.05	0.09	0.23	0.16	0.07	0.22	0.03	0.13	0.09	−0.01	−0.02	0.05	0.02
**Suzuki Beta-cell - Proinsulin**	0.27	1.00	−0.07	0.00	0.01	−0.03	0.03	0.01	0.03	0.26	0.24	−0.03	0.09	0.15	0.09	0.01	0.01	0.01	0.02	−0.02	0.00	0.00	0.05	0.11	0.04	−0.02	0.02	0.02	0.02
**Suzuki Beta-cell + Proinsulin**	0.45	−0.07	1.00	0.05	−0.02	0.00	0.02	−0.05	−0.01	0.22	0.40	0.13	0.04	0.20	0.55	0.00	−0.01	0.02	0.00	0.02	−0.01	−0.08	−0.01	0.02	0.05	−0.01	−0.04	0.01	0.03
**Suzuki Body fat**	0.38	0.00	0.05	1.00	−0.02	−0.01	0.07	−0.01	0.04	0.00	0.11	0.01	0.15	0.04	0.13	−0.03	−0.03	−0.01	−0.04	−0.02	−0.04	0.03	0.00	−0.04	0.05	0.00	−0.04	0.02	0.03
**Suzuki Lipodystrophy**	0.24	0.01	−0.02	−0.02	1.00	−0.02	0.04	−0.02	0.02	0.01	0.27	0.05	0.20	0.04	0.02	−0.04	−0.02	0.18	0.62	0.31	−0.01	−0.02	−0.05	0.11	0.04	0.00	0.02	0.08	0.01
**Suzuki Liver and lipid metabolism**	0.07	−0.03	0.00	−0.01	−0.02	1.00	0.04	0.04	0.01	0.01	0.09	−0.03	0.00	0.03	0.01	−0.01	0.56	−0.01	−0.02	0.34	0.02	0.06	−0.01	−0.01	−0.06	0.01	0.04	0.05	−0.01
**Suzuki Metabolic syndrome**	0.41	0.03	0.02	0.07	0.04	0.04	1.00	0.00	−0.03	0.10	0.28	0.01	0.18	0.16	0.09	0.01	0.02	−0.02	0.19	0.11	−0.01	0.00	0.05	0.04	0.00	−0.02	0.00	0.02	−0.02
**Suzuki Obesity**	0.36	0.01	−0.05	−0.01	−0.02	0.04	0.00	1.00	0.00	0.10	0.26	0.03	0.01	0.10	0.02	0.02	0.06	0.06	−0.02	0.04	0.05	0.57	0.03	0.02	0.03	0.03	0.02	0.00	−0.02
**Suzuki Residual glycemic**	0.40	0.03	−0.01	0.04	0.02	0.01	−0.03	0.00	1.00	0.11	0.17	0.00	−0.02	0.16	0.03	0.07	0.04	0.06	0.05	0.02	0.19	0.03	−0.01	0.10	0.04	0.02	−0.02	0.01	0.01
**Smith Proinsulin**	0.32	0.26	0.22	0.00	0.01	0.01	0.10	0.10	0.11	1.00	0.45	0.14	0.02	0.39	0.22	0.04	0.03	0.05	0.01	0.00	0.01	0.12	0.02	0.11	0.04	−0.04	0.00	−0.03	−0.05
**Smith Global PRS**	0.67	0.24	0.40	0.11	0.27	0.09	0.28	0.26	0.17	0.45	1.00	0.10	0.26	0.47	0.49	0.05	0.06	0.25	0.32	0.24	0.11	0.31	0.06	0.20	0.07	0.00	−0.02	0.03	0.01
**Smith ALP negative**	0.08	−0.03	0.13	0.01	0.05	−0.03	0.01	0.03	0.00	0.14	0.10	1.00	0.02	0.06	0.06	0.02	−0.03	−0.03	0.07	0.03	0.07	0.00	−0.04	0.00	0.00	−0.04	0.06	0.00	−0.01
**Smith ASAT positive**	0.20	0.09	0.04	0.15	0.20	0.00	0.18	0.01	−0.02	0.02	0.26	0.02	1.00	0.05	0.05	−0.02	−0.04	0.07	0.26	0.09	−0.02	0.02	−0.02	0.02	0.05	0.02	−0.01	0.01	−0.04
**Smith Beta Cell 1**	0.33	0.15	0.20	0.04	0.04	0.03	0.16	0.10	0.16	0.39	0.47	0.06	0.05	1.00	0.21	0.07	0.03	−0.02	0.05	0.01	0.05	0.08	0.03	0.00	0.08	−0.01	0.00	−0.06	−0.04
**Smith Beta Cell 2**	0.39	0.09	0.55	0.13	0.02	0.01	0.09	0.02	0.03	0.22	0.49	0.06	0.05	0.21	1.00	0.02	0.01	−0.01	0.04	0.01	0.07	−0.02	−0.02	0.06	0.06	−0.01	−0.05	−0.01	0.05
**Smith Bilirubin**	0.02	0.01	0.00	−0.03	−0.04	−0.01	0.01	0.02	0.07	0.04	0.05	0.02	−0.02	0.07	0.02	1.00	−0.02	−0.02	−0.04	−0.01	0.05	−0.02	0.00	−0.04	0.04	−0.05	0.03	−0.02	0.00
**Smith Cholesterol**	0.05	0.01	−0.01	−0.03	−0.02	0.56	0.02	0.06	0.04	0.03	0.06	−0.03	−0.04	0.03	0.01	−0.02	1.00	−0.02	−0.03	0.02	0.06	0.08	−0.04	−0.01	−0.07	−0.01	0.06	0.01	−0.01
**Smith Hyper Insulin**	0.09	0.01	0.02	−0.01	0.18	−0.01	−0.02	0.06	0.06	0.05	0.25	−0.03	0.07	−0.02	−0.01	−0.02	−0.02	1.00	0.14	0.13	0.03	0.06	0.05	0.07	0.04	0.03	−0.02	0.05	0.00
**Smith Lipodystrophy 1**	0.23	0.02	0.00	−0.04	0.62	−0.02	0.19	−0.02	0.05	0.01	0.32	0.07	0.26	0.05	0.04	−0.04	−0.03	0.14	1.00	0.20	−0.01	−0.02	0.05	0.03	0.04	−0.03	0.02	0.01	0.00
**Smith Lipodystrophy 2**	0.16	−0.02	0.02	−0.02	0.31	0.34	0.11	0.04	0.02	0.00	0.24	0.03	0.09	0.01	0.01	−0.01	0.02	0.13	0.20	1.00	0.01	−0.03	0.04	0.20	0.03	0.02	−0.02	0.06	0.02
**Smith Liver-Lipid**	0.07	0.00	−0.01	−0.04	−0.01	0.02	−0.01	0.05	0.19	0.01	0.11	0.07	−0.02	0.05	0.07	0.05	0.06	0.03	−0.01	0.01	1.00	0.01	0.02	−0.01	−0.02	−0.05	0.06	−0.04	0.00
**Smith Obesity**	0.22	0.00	−0.08	0.03	−0.02	0.06	0.00	0.57	0.03	0.12	0.31	0.00	0.02	0.08	−0.02	−0.02	0.08	0.06	−0.02	−0.03	0.01	1.00	0.00	0.01	0.03	0.05	−0.02	0.04	0.00
**Smith SHBG-LpA**	0.03	0.05	−0.01	0.00	−0.05	−0.01	0.05	0.03	−0.01	0.02	0.06	−0.04	−0.02	0.03	−0.02	0.00	−0.04	0.05	0.05	0.04	0.02	0.00	1.00	0.15	−0.01	0.02	0.01	−0.02	−0.02
**Smith VAT negative**	0.13	0.11	0.02	−0.04	0.11	−0.01	0.04	0.02	0.10	0.11	0.20	0.00	0.02	0.00	0.06	−0.04	−0.01	0.07	0.03	0.20	−0.01	0.01	0.15	1.00	0.01	0.01	−0.04	−0.02	0.01
**MRS**	0.09	0.04	0.05	0.05	0.04	−0.06	0.00	0.03	0.04	0.04	0.07	0.00	0.05	0.08	0.06	0.04	−0.07	0.04	0.04	0.03	−0.02	0.03	−0.01	0.01	1.00	−0.10	0.03	−0.03	−0.08

S. Figure 3: Restricted cubic spline for the association between DII and type 2 diabetes

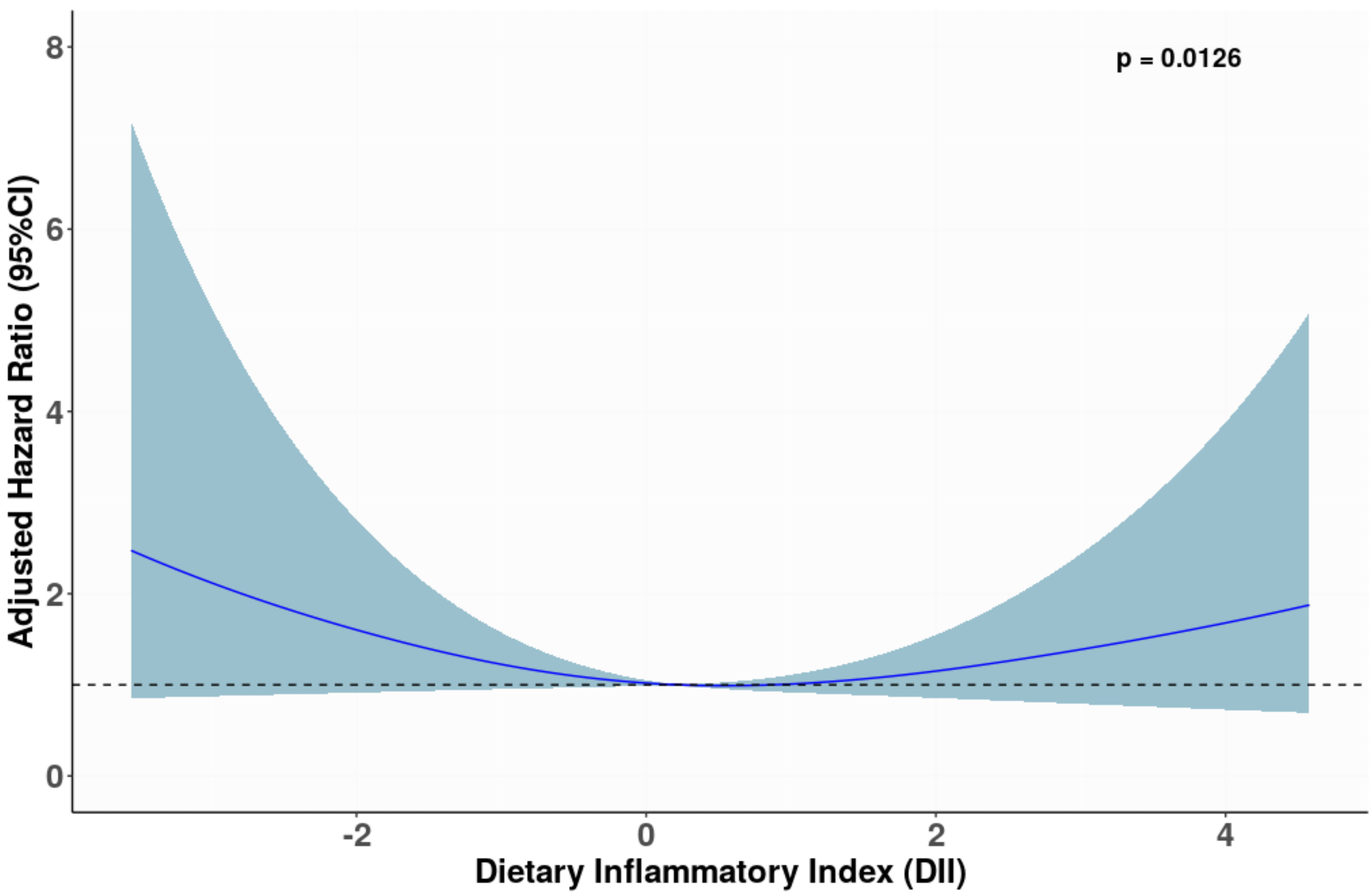

Model adjusted for age (as underlying time variable), sex, education, physical activity and smoking.
Knot placement was at 10th, 50th, and 90th percentile. Reference point is median DII in the subcohort. P value represents the test for nonlinearity results

S. Figure 4: Association between the polygenic risk scores (per 1 SD increment) and incident type 2 diabetes

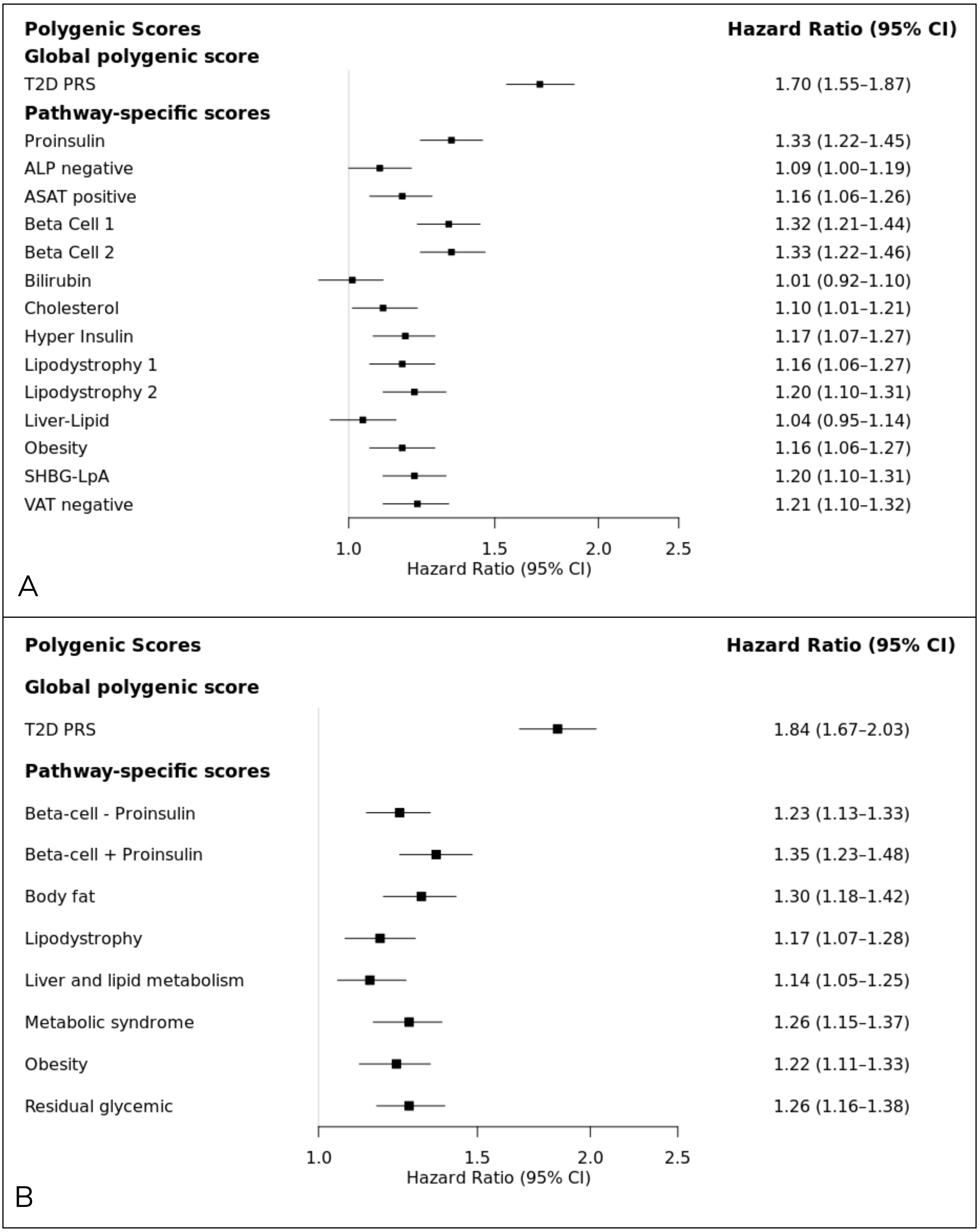

Association between type 2 diabetes and 1 SD increment of soft clustering-derived (A) and hard clustering-derived (B) polygenic risk scores. Model adjusted for age (as underlying time variable) and sex.

S. Figure 5: Association between the polygenic risk scores (per 1 SD increment) and incident type 2 diabetes after excluding prevalent CVD cases

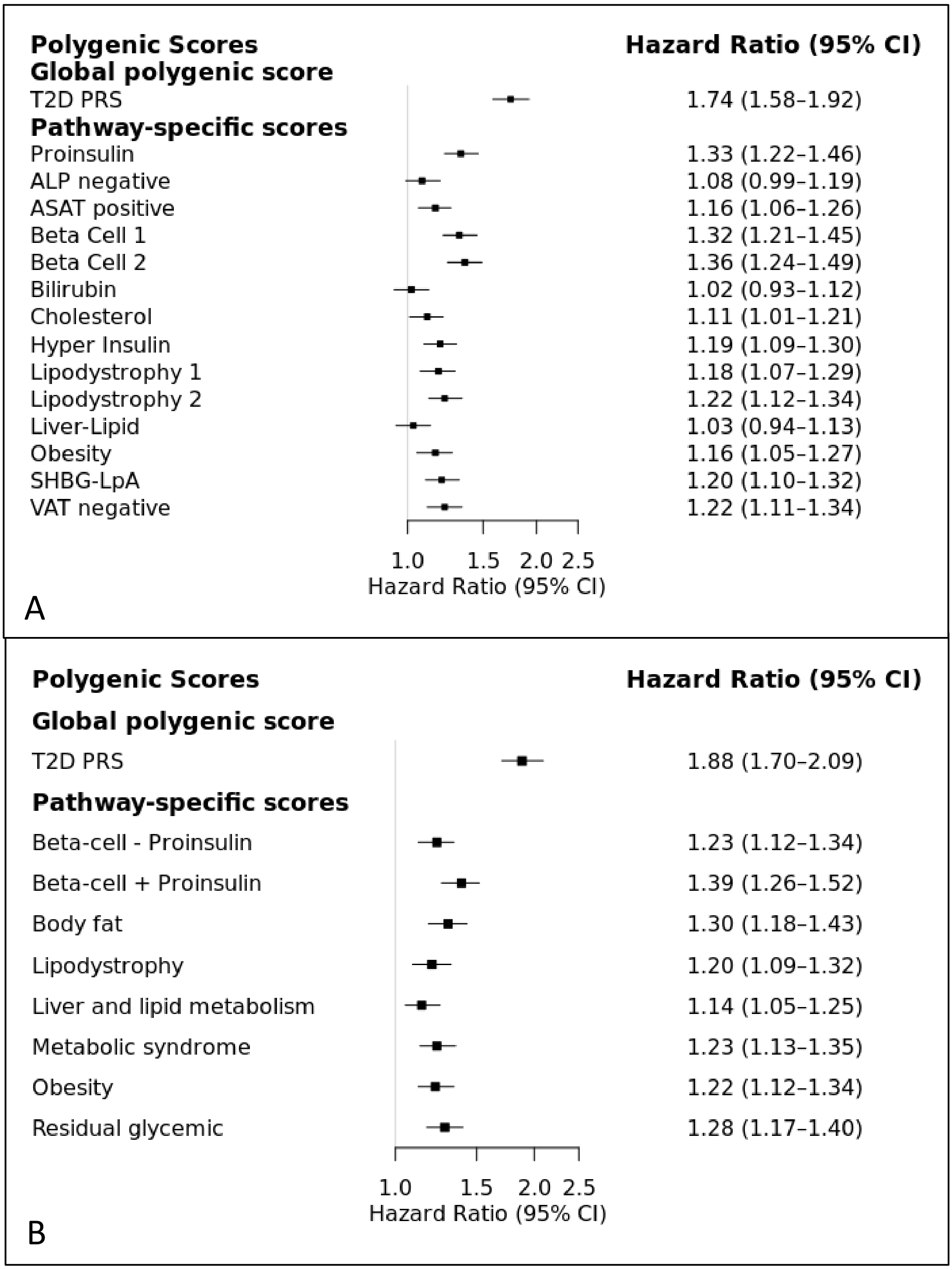

Association between type 2 diabetes and 1 SD increment of soft clustering-derived (A) and hard clustering-derived (B) polygenic risk scores. Model adjusted for age (as underlying time variable) and sex.

S. Table 1: Composition of the assessed DQIs

DQIs	Components	Food items	Scoring criteria	
			Criteria for minimum score (0)	Criteria for maximum score (10)
**AHEI-2010**	Vegetables, servings/d	Raw vegetables, cabbage, cooked vegetables, mushrooms, garlic	0	≥5
	Fruits, servings/d	Fresh fruits	0	≥4
	Whole grains, g/d	Whole grain bread, grainflakes, grains, muesli	0	75 for women, 90 for men
	Sugar-sweetened beverages and fruit juice, servings/d	Low-energy soft drinks, high-energy soft drinks, fruit juice	≥1	0
	Nuts and legumes, servings/d	Nuts and legumes (green peas, green beans, lentil, peas, bean stew)	0	≥1
	Red/processed meat, servings/d	Red and processed meats	≥1.5	0
	Long-chain fatty acis (EPA+DHA), mg/d	_	0	250
	PUFA, % of energy	_	≤2	≥10
	Alcohol, drinks/d[1]	Beer, wine, fruitwine, sparkling wine, aperitif, spirits	≥2.5 for women, ≥3.5 for men	0.5-1.5 for women, 0.5-2 for men
**DASH**	Fruits	Fresh fruit, canned fruit, fruit juice	Q1 = 1 point , Q2 = 2 points , Q3 = 3 points , Q4 = 4 points , Q5 = 5 points	
	Vegetables	Raw vegetables, cabbage, cooked vegetables, mushrooms, garlic		
	Nuts and legumes	Nuts and legumes (green peas, green beans, lentil, peas, bean stew)		
	Whole grains	Whole grain bread, grainflakes, grains, muesli		
	Low-fat dairy	Low-fat dairy, low-fat cheese		
	Red and processed meats	Red and processed meats	Q1 = 5 points , Q2 = 4 points, Q3 = 3 points, Q4 = 2 points, Q5 = 1 point	
	Sweetened beverages	Low-energy soft drinks, high-energy soft drinks		

			Criteria for minimum score (0)	Criteria for maximum score (1)
**MedPyr**	Vegetables, servings/d[2]	Raw vegetables, cabbage, cooked vegetables, mushrooms, garlic	0	≥ 6
	Legumes, serving/week[2]	Green peas, green beans, lentil, peas, bean stew	0	≥ 2
	Fruits, servings/d[3]	Fresh fruits	0	3–6
	Nuts, servings/d[3]	Nuts	0	1–2
	Cereals, servings/d[3]	Whole grain bread, other bread, grainflakes, grains, muesli, cornflakes, crisps, pasta, rice	0	3–6
	Dairy, servings/d[3]	Milk, yogurt, fruit yogurt, curd, kefir, whipped cream, low fat cheese, high fat cheese	0	1.5–2.5
	Fish, servings/week[2]	Fish (preserved and smoked is also considered)	0	≥ 2
	Red meat, servings/week[4]	Red meat	≥ 4	< 2
	Processed meat, servings/week[4]	Processed meat	≥ 2	≤ 1
	White meat, servings/week[3]	Poultry	0	1.5–2.5
	Eggs, servings/week[3]	Eggs	0	2–4
	Potatoes, servings/week[4]	Potatoes and fried potatoes	≥ 6	≤ 3
	Alcohol, g/d[5]	Overall alcohol intake	> 50 for men, > 25 for women	10–50 for men, 5–25 for women
	Sweets, servings/week[4]	Cakes, cookies, confectionnary, desserts	≥ 4	≤ 2
	Olive oil, g/d	Olive oil for salad dressing, preparation of vegetables and preparation of meat	Non-consumers	Consumers
**DII**	Energy (kcal)		Centered percentile value for each food parameter was calculated based on dietary intake data and world average and standard deviation. Each parameter-specific value was then multiplied by its corresponding literature-derived inflammatory effect score to obtain the food parameter-specific score, which were then summed up to calculate the overall DII score.	
	Carbohydrate (g)			
	Protein (g)			
	Total fat (g)			
	Alcohol (g)			
	Fibre (g)			
	Cholesterol (mg)			
	Saturated fat (g)			
	MUFA (g)			

	PUFA (g)	
	n-3 fatty acids (g)	
	n-6 fatty acids (g)	
	Niacin (mg)	
	Thiamin (mg)	
	Riboflavin (mg)	
	Vitamin B12 (µg)	
	Vitamin B6 (mg)	
	Fe (mg)	
	Mg (mg)	
	Zn (mg)	
	Vitamin A (RE)	
	Vitamin C (mg)	
	Vitamin D (µg)	
	Vitamin E (mg)	
	Folic acid (µg)	
	B-carotene (µg)	
	Garlic (g)	
	Onion (g)	
	Tea (g): black tea, green tea, fruit tea, herbal tea (pure, with milk, with condensed milk, with sweetener, with lemon juice)	

[1]: Non-drinkers received a score of 2.5

[2]: For components for which a high consumption was recommended, continuous scores from 0 to 1 were assigned proportionally from no consumption to meeting the recommended level of consumption

[3]: For components for which moderate consumption was recommended, we assigned a score of 1 for consumption within the recommended levels and 0 for no consumption, with consumption levels in between scored proportionately; overconsumption (double the mid-point value of the recommended intake) was penalized and received a maximum score of 0.5, with consumption between the recommended level and the penalty point scored proportionally

[4]: For components for which a low consumption was recommended, consumption below the recommended levels was assigned a score of 1 and double the recommended levels were assigned a score of 0; levels in between scored proportionally

[5]: For alcohol, we assigned a score of 1 for consumption levels within recommendation, non-consumption was scored 0.5 while overconsumption was scored 0. Intake between 0 and the minimum level of the recommended range received a score of 0.75

S. Table 2: Baseline characteristics of the analytical sample for the PRS analyses

**Characteristics**	**Subcohort**[1]	**Cases**[1]
Sex		
Male	836 (38%)	395 (58%)
Female	1,368 (62%)	281 (42%)
Age, years	49.6 (42.1, 57.8)	56.5 (49.5, 60.9)
Waist circumference, cm	85.0 (75.1, 94.0)	100.0 (92.0, 107.5)
BMI, kg/m²	25.5 (23.1, 28.2)	29.8 (27.2, 32.8)
Smoking status		
Never-smoker	1,051 (48%)	237 (35%)
Smoker	452 (21%)	140 (21%)
Former smoker	701 (32%)	299 (44%)
Educational status		
Technical college	539 (24%)	163 (24%)
University	851 (39%)	211 (31%)
No/vocational training	814 (37%)	302 (45%)
Physical activity, h/wk	4.5 (2.0, 8.0)	4.5 (1.5, 8.5)
Alcohol intake, g/day	8.2 (2.7, 19.6)	7.9 (2.4, 20.5)
Prevalent hypertension	1,046 (47%)	520 (77%)
Prevalent dyslipidemia	585 (27%)	290 (43%)
Total energy intake, kcal/day	2,004 (1,630, 2,472)	2,083 (1,696, 2,474)
DASH	21.3 (4.4)	21.1 (4.3)
DII	0.3 (1.7)	0.2 (1.7)
AHEI 2010	37.0 (8.7)	35.9 (8.3)
MedPyr	7.0 (1.1)	6.9 (1.1)

[1]n (%); Median (Q1, Q3); Mean (SD). AHEI: Alternative Healthy Eating Index. DASH: Dietary Approaches to Stop Hypertension. DII: Dietary Inflammatory Index. MedPyr: Mediterranean Diet Pyramid.

S. Table 3: Baseline characteristics of the analytical sample for MRS analyses

**Characteristics**	**Subcohort**[1]	**Cases**[1]
Sex		
Male	416 (39%)	365 (57%)
Female	649 (61%)	281 (43%)
Age, years	50.3 (42.3, 57.9)	56.6 (49.5, 60.8)
Waist circumference, cm	85.0 (75.0, 93.5)	99.5 (91.5, 106.5)
BMI, kg/m²	25.5 (23.1, 28.1)	29.8 (27.2, 32.4)
Smoking status		
Never-smoker	519 (49%)	219 (34%)
Smoker	216 (20%)	132 (20%)
Former smoker	330 (31%)	295 (46%)
Educational status		
Technical college	253 (24%)	155 (24%)
University	395 (37%)	198 (31%)
No/vocational training	417 (39%)	293 (45%)
Physical activity, h/wk	5.0 (2.0, 8.0)	4.5 (1.5, 8.5)
Alcohol intake, g/day	8.4 (2.9, 20.1)	8.0 (2.5, 20.4)
Prevalent hypertension	541 (51%)	499 (77%)
Prevalent dyslipidemia	289 (27%)	289 (45%)
Total energy intake, kcal/day	2,011 (1,620, 2,437)	2,074 (1,685, 2,480)
DASH	21.3 (4.5)	21.1 (4.3)
DII	0.3 (1.7)	0.2 (1.7)
AHEI 2010	37.2 (8.8)	35.9 (8.5)
MedPyr	7.1 (1.1)	6.9 (1.1)

[1]n (%); Median (Q1, Q3); Mean (SD).). AHEI: Alternative Healthy Eating Index. DASH: Dietary Approaches to Stop Hypertension. DII: Dietary Inflammatory Index. MedPyr: Mediterranean Diet Pyramid.

S. Table 4: Association between the DQIs and incident type 2 diabetes, modelled continuously and per extreme quintile contrast

	Model	HR (95% CI)
**AHEI-2010**		
Per 1 SD increase	Model 1	0.88 (0.81-0.96)
	Model 2	0.92 (0.84-1.00)
Q5 vs. Q1 contrast	Model 1	0.65 (0.49-0.86)
	Model 2	0.73 (0.54-0.98)
**DASH**		
Per 1 SD increase	Model 1	0.90 (0.83-0.99)
	Model 2	0.95 (0.86-1.04)
Q5 vs. Q1 contrast	Model 1	0.75 (0.56-1.00)
	Model 2	0.85 (0.63-1.14)
**DII**		
Per 1 SD increase	(non-linear)	
Q5 vs. Q1 contrast	Model 1	1.18 (0.89-1.55)
	Model 2	1.08 (0.81-1.45)
**MedPyr**		
Per 1 SD increase	Model 1	0.96 (0.88-1.05)
	Model 2	1.02 (0.93-1.11)
Q5 vs. Q1 contrast	Model 1	0.86 (0.65-1.13)
	Model 2	0.99 (0.74-1.32)

Model 1: age (as underlying time variable) and sex.
Model 2: model 1 + education, physical activity, smoking status, energy intake (except DII), alcohol intake (DASH only).
AHEI: Alternative Healthy Eating Index. DASH: Dietary Approaches to Stop Hypertension. DII: Dietary Inflammatory Index. MedPyr: Mediterranean Diet Pyramid.

S. Table 5: Association between the DQIs and incident type 2 diabetes, modelled continuously and per extreme quintile contrast after excluding prevalent CVD cases

	Model	HR (95%CI)
**AHEI-2010**		
Per 1 SD increase	Model 1	0.88 (0.81-0.96)
	Model 2	0.92 (0.84-1.01)
Q5 vs. Q1 contrast	Model 1	0.66 (0.49-0.88)
	Model 2	0.74 (0.55-1.00)
**DASH**		
Per 1 SD increase	Model 1	0.90 (0.82-0.98)
	Model 2	0.94 (0.86-1.04)
Q5 vs. Q1 contrast	Model 1	0.71 (0.53-0.95)
	Model 2	0.81 (0.60-1.11)
**DII**		
Per 1 SD increase	– (non linear)	
Q5 vs. Q1 contrast	Model 1	1.16 (0.87-1.54)
	Model 2	1.08 (0.80-1.44)
**MedPyr**		
Per 1 SD increase	Model 1	0.96 (0.88-1.05)
	Model 2	1.02 (0.93-1.11)
Q5 vs. Q1 contrast	Model 1	0.87 (0.65-1.16)
	Model 2	1.01 (0.75-1.36)

Model 1: age (as underlying time variable) and sex.
Model 2: model 1 + education, physical activity, smoking status, energy intake (except DII), alcohol intake (DASH only).
AHEI: Alternative Healthy Eating Index. DASH: Dietary Approaches to Stop Hypertension. DII: Dietary Inflammatory Index. MedPyr: Mediterranean Diet Pyramid.

S. Table 6: Association between the assessed DQIs and incident type 2 diabetes across tertiles of MRS

DQI	MRS	HR (95% CI)	p-value
**AHEI-2010**	Low	0.46 (0.21 - 1.02)	0.055
	Medium	0.58 (0.33 - 1.00)	0.049
	High	1.02 (0.71 - 1.48)	0.897
**DASH**	Low	0.32 (0.12 - 0.81)	0.016
	Medium	0.78 (0.44 - 1.37)	0.384
	High	1.53 (1.04 - 2.27)	0.032
**DII**	Low	2.44 (1.12 - 5.33)	0.025
	Medium	0.81 (0.48 - 1.36)	0.424
	High	0.94 (0.65 - 1.34)	0.721
**MedPyr**	Low	0.60 (0.29 - 1.21)	0.153
	Medium	0.89 (0.54 - 1.48)	0.654
	High	1.18 (0.82 - 1.71)	0.371

Model adjusted for age (as underlying time variable), sex, proportions of B lymphocytes, CD4+ T lymphocytes, CD8+ T lymphocytes, monocytes, and natural killer (NK) cells, education, physical activity, smoking status, energy intake (except DII), alcohol intake (DASH only). AHEI: Alternative Healthy Eating Index. DASH: Dietary Approaches to Stop Hypertension. DII: Dietary Inflammatory Index. MedPyr: Mediterranean Diet Pyramid.

S. Table 7: P-interaction values for DQIxMRS in main and sensitivity analyses

**DQI x MRS**	**Model**	**P interaction***
AHEI-2010	Model 1	**0.008**
	Model 2	**0.012**
	Model 3	**0.001**
	Model 4	**0.019**
DASH	Model 1	0.348
	Model 2	0.283
	Model 3	0.244
	Model 4	0.549
MedPyr	Model 1	**< 0.0001**
	Model 2	**< 0.0001**
	Model 3	**0.001**
	Model 4	0.532
DII	Model 1	**0.01**
	Model 2	**0.003**
	Model 3	0.109
	Model 4	0.180

Model 1 (main): adjusted for age (as underlying time variable), sex, proportions of B lymphocytes, CD4+ T lymphocytes, CD8+ T lymphocytes, monocytes, and natural killer (NK) cells, education, physical activity, smoking status, energy intake (except DII), alcohol intake (DASH only).
Model 2: as model 1, in sample excluding prevalent CVD cases
Model 3: as model 1, in sample excluding participants with baseline HbA1c ≥6.5%
Model 4: as model 1, additionally adjusted for BMI, HbA1c, TG and HDL.
*p-interaction (continuous DQI*continuous MRS) in the case of AHEI-2010, DASH and MedPyr. For DII, p-values were derived from likelihood ratio tests comparing models with and without the interaction term (DII categorical as tertile x continuous MRS).

S. Table 8: Association between the assessed DQIs and incident type 2 diabetes across tertiles of genetic risk defined by soft clustering-derived scores

DQI	PRS/pPRS	Risk Subgroup	HR (95% CI)	p-value	p-interaction*
**AHEI-2010**	ALP negative	Low	0.71 (0.47-1.07)	0.103	0.997
		Medium	0.81 (0.55-1.20)	0.301	
		High	0.76 (0.52-1.12)	0.172	
	ASAT positive	Low	0.63 (0.42-0.93)	0.019	0.997
		Medium	1.04 (0.70-1.54)	0.847	
		High	0.78 (0.53-1.15)	0.205	
	Beta Cell 1	Low	0.80 (0.52-1.23)	0.310	0.9
		Medium	0.59 (0.39-0.87)	0.009	
		High	1.04 (0.72-1.49)	0.849	
	Beta Cell 2	Low	1.10 (0.68-1.79)	0.685	0.997
		Medium	0.53 (0.35-0.79)	0.002	
		High	0.81 (0.57-1.16)	0.252	
	Bilirubin	Low	0.66 (0.46-0.95)	0.023	0.997
		Medium	0.85 (0.59-1.20)	0.353	
		High	0.67 (0.40-1.14)	0.141	
	Cholesterol	Low	0.72 (0.49-1.06)	0.097	0.997
		Medium	0.76 (0.50-1.15)	0.189	
		High	0.88 (0.60-1.29)	0.518	
	Global PRS	Low	0.80 (0.48-1.33)	0.387	0.997
		Medium	0.74 (0.48-1.15)	0.182	
		High	0.83 (0.60-1.15)	0.257	
	Hyper Insulin	Low	0.94 (0.61-1.44)	0.782	0.997
		Medium	0.80 (0.53-1.20)	0.279	
		High	0.70 (0.49-0.98)	0.041	
	Lipodystrophy 1	Low	0.74 (0.50-1.10)	0.141	0.9
		Medium	0.73 (0.49-1.10)	0.130	
		High	0.87 (0.60-1.26)	0.452	
	Lipodystrophy 2	Low	0.72 (0.47-1.12)	0.148	0.9
		Medium	0.91 (0.62-1.33)	0.625	
		High	0.66 (0.45-0.96)	0.028	
	Liver-Lipid	Low	0.74 (0.48-1.13)	0.161	0.993
		Medium	0.85 (0.58-1.25)	0.407	
		High	0.64 (0.43-0.96)	0.031	
	Obesity	Low	0.58 (0.38-0.91)	0.017	0.727
		Medium	0.68 (0.45-1.02)	0.063	
		High	0.98 (0.69-1.40)	0.904	
	Proinsulin	Low	0.52 (0.33-0.83)	0.006	0.9
		Medium	1.00 (0.67-1.49)	0.999	
		High	0.91 (0.64-1.30)	0.609	
	SHBG-LpA	Low	0.67 (0.43-1.03)	0.065	0.997
		Medium	0.85 (0.57-1.26)	0.422	
		High	0.77 (0.53-1.11)	0.166	
	VAT negative	Low	0.67 (0.43-1.03)	0.071	0.997
		Medium	0.99 (0.67-1.45)	0.948	
		High	0.67 (0.46-0.98)	0.041	

**DASH**	ALP negative	Low	0.60 (0.40-0.92)	0.018	0.355
		Medium	0.77 (0.50-1.18)	0.223	
		High	1.19 (0.81-1.76)	0.382	
	ASAT positive	Low	0.92 (0.60-1.40)	0.688	0.561
		Medium	0.94 (0.62-1.43)	0.776	
		High	0.80 (0.54-1.18)	0.256	
	Beta Cell 1	Low	0.99 (0.63-1.56)	0.972	0.882
		Medium	0.77 (0.50-1.16)	0.211	
		High	0.92 (0.64-1.30)	0.622	
	Beta Cell 2	Low	0.88 (0.53-1.46)	0.615	0.882
		Medium	0.88 (0.60-1.29)	0.522	
		High	0.97 (0.67-1.41)	0.893	
	Bilirubin	Low	0.70 (0.48-1.00)	0.052	0.355
		Medium	0.90 (0.63-1.30)	0.588	
		High	1.52 (0.81-2.84)	0.188	
	Cholesterol	Low	0.91 (0.61-1.35)	0.640	0.882
		Medium	0.71 (0.47-1.09)	0.120	
		High	1.13 (0.75-1.69)	0.564	
	Global PRS	Low	1.02 (0.61-1.70)	0.935	0.735
		Medium	0.69 (0.44-1.08)	0.103	
		High	0.98 (0.71-1.37)	0.914	
	Hyper Insulin	Low	1.33 (0.87-2.03)	0.184	0.467
		Medium	0.90 (0.59-1.38)	0.632	
		High	0.65 (0.45-0.93)	0.019	
	Lipodystrophy 1	Low	0.99 (0.64-1.52)	0.946	0.882
		Medium	1.00 (0.67-1.48)	0.986	
		High	0.82 (0.55-1.22)	0.336	
	Lipodystrophy 2	Low	0.93 (0.61-1.40)	0.720	0.355
		Medium	1.11 (0.72-1.69)	0.644	
		High	0.70 (0.47-1.04)	0.075	
	Liver-Lipid	Low	0.64 (0.40-1.01)	0.053	0.467
		Medium	1.00 (0.69-1.45)	0.991	
		High	1.13 (0.76-1.67)	0.557	
	Obesity	Low	0.76 (0.49-1.17)	0.216	0.897
		Medium	0.82 (0.54-1.24)	0.345	
		High	1.11 (0.76-1.61)	0.596	
	Proinsulin	Low	0.77 (0.50-1.20)	0.253	0.467
		Medium	0.80 (0.52-1.22)	0.296	
		High	1.14 (0.79-1.64)	0.489	
	SHBG-LpA	Low	0.74 (0.49-1.13)	0.162	0.467
		Medium	0.93 (0.63-1.38)	0.720	
		High	1.15 (0.77-1.73)	0.496	
	VAT negative	Low	0.78 (0.51-1.20)	0.260	0.882
		Medium	0.97 (0.65-1.45)	0.880	
		High	1.01 (0.70-1.46)	0.941	
**DII**	ALP negative	Low	1.51 (1.00-2.27)	0.049	0.476
		Medium	0.96 (0.65-1.42)	0.856	
		High	0.99 (0.67-1.46)	0.940	
	ASAT positive	Low	1.14 (0.75-1.74)	0.540	0.738

		Medium	0.89 (0.60-1.32)	0.553	
		High	1.19 (0.82-1.70)	0.359	
	Beta Cell 1	Low	1.16 (0.75-1.79)	0.517	0.896
		Medium	1.44 (0.96-2.16)	0.075	
		High	0.93 (0.65-1.33)	0.696	
	Beta Cell 2	Low	0.95 (0.60-1.51)	0.826	0.643
		Medium	1.58 (1.07-2.34)	0.021	
		High	0.85 (0.59-1.22)	0.377	
	Bilirubin	Low	1.15 (0.82-1.62)	0.409	0.476
		Medium	1.21 (0.84-1.74)	0.308	
		High	0.71 (0.41-1.22)	0.216	
	Cholesterol	Low	1.32 (0.91-1.93)	0.145	0.476
		Medium	0.99 (0.66-1.49)	0.974	
		High	0.84 (0.57-1.25)	0.400	
	Global PRS	Low	0.96 (0.58-1.59)	0.873	0.643
		Medium	1.12 (0.70-1.78)	0.633	
		High	1.16 (0.84-1.61)	0.362	
	Hyper Insulin	Low	0.79 (0.53-1.19)	0.263	0.643
		Medium	1.45 (0.96-2.20)	0.078	
		High	1.09 (0.77-1.55)	0.633	
	Lipodystrophy 1	Low	0.86 (0.57-1.30)	0.468	0.643
		Medium	1.03 (0.70-1.52)	0.887	
		High	1.37 (0.94-2.01)	0.104	
	Lipodystrophy 2	Low	0.99 (0.65-1.50)	0.947	0.638
		Medium	1.13 (0.76-1.67)	0.543	
		High	1.08 (0.74-1.57)	0.706	
	Liver-Lipid	Low	1.52 (1.00-2.31)	0.051	0.476
		Medium	0.96 (0.66-1.41)	0.854	
		High	0.89 (0.61-1.31)	0.563	
	Obesity	Low	0.89 (0.57-1.37)	0.590	0.476
		Medium	1.22 (0.82-1.82)	0.326	
		High	1.14 (0.79-1.63)	0.491	
	Proinsulin	Low	1.23 (0.80-1.89)	0.343	0.669
		Medium	0.95 (0.62-1.44)	0.793	
		High	1.12 (0.80-1.58)	0.518	
	SHBG-LpA	Low	1.13 (0.74-1.72)	0.584	0.738
		Medium	1.10 (0.75-1.62)	0.623	
		High	0.84 (0.57-1.24)	0.378	
	VAT negative	Low	1.00 (0.66-1.52)	1.000	0.643
		Medium	0.83 (0.57-1.22)	0.352	
		High	1.40 (0.95-2.04)	0.086	
**MedPyr**	ALP negative	Low	1.18 (0.79-1.76)	0.413	0.968
		Medium	0.95 (0.66-1.36)	0.775	
		High	1.08 (0.73-1.59)	0.695	
	ASAT positive	Low	1.01 (0.68-1.49)	0.967	0.968
		Medium	1.24 (0.82-1.85)	0.308	
		High	1.09 (0.77-1.56)	0.620	
	Beta Cell 1	Low	1.30 (0.86-1.96)	0.212	0.968
		Medium	0.98 (0.66-1.47)	0.941	

		High	1.03 (0.72-1.48)	0.866	
	Beta Cell 2	Low	1.39 (0.89-2.18)	0.152	0.968
		Medium	0.97 (0.65-1.44)	0.881	
		High	1.00 (0.71-1.41)	0.984	
	Bilirubin	Low	0.82 (0.59-1.15)	0.255	0.968
		Medium	1.23 (0.87-1.74)	0.245	
		High	1.54 (0.83-2.88)	0.174	
	Cholesterol	Low	1.08 (0.75-1.55)	0.673	0.968
		Medium	1.15 (0.77-1.72)	0.497	
		High	1.06 (0.71-1.60)	0.771	
	Global PRS	Low	1.34 (0.83-2.17)	0.227	0.968
		Medium	0.75 (0.50-1.13)	0.170	
		High	1.13 (0.81-1.58)	0.456	
	Hyper Insulin	Low	1.21 (0.81-1.81)	0.360	0.968
		Medium	0.94 (0.61-1.43)	0.767	
		High	0.91 (0.64-1.30)	0.594	
	Lipodystrophy 1	Low	1.22 (0.82-1.81)	0.333	0.968
		Medium	0.95 (0.64-1.40)	0.789	
		High	1.17 (0.81-1.68)	0.399	
	Lipodystrophy 2	Low	1.07 (0.72-1.59)	0.747	0.968
		Medium	1.01 (0.70-1.48)	0.941	
		High	1.08 (0.74-1.57)	0.695	
	Liver-Lipid	Low	0.89 (0.60-1.33)	0.582	0.968
		Medium	1.08 (0.72-1.61)	0.724	
		High	1.20 (0.83-1.73)	0.332	
	Obesity	Low	1.07 (0.69-1.66)	0.754	0.968
		Medium	0.96 (0.66-1.39)	0.821	
		High	0.98 (0.68-1.42)	0.914	
	Proinsulin	Low	0.74 (0.47-1.17)	0.198	0.968
		Medium	1.56 (1.05-2.33)	0.027	
		High	0.98 (0.70-1.37)	0.896	
	SHBG-LpA	Low	1.40 (0.94-2.08)	0.094	0.968
		Medium	0.99 (0.68-1.45)	0.959	
		High	0.91 (0.62-1.35)	0.644	
	VAT negative	Low	1.17 (0.77-1.77)	0.458	0.968
		Medium	0.92 (0.64-1.31)	0.632	
		High	1.28 (0.87-1.87)	0.209	

Model adjusted for age (as underlying time variable), sex, education, physical activity, smoking status, energy intake (except DII), alcohol intake (DASH only).
AHEI: Alternative Healthy Eating Index. DASH: Dietary Approaches to Stop Hypertension. DII: Dietary Inflammatory Index. MedPyr: Mediterranean Diet Pyramid.
*p-interaction (continuous DQI*continuous PRS or pPRS) FDR-adjusted in the case of AHEI-2010, DASH and MedPyr. For DII, p-values were derived from likelihood ratio tests comparing models with and without the interaction term (DII categorical as tertile x continuous PRS), and p-values were FDR adjusted.

S. Table 9: Association between the assessed DQIs and incident type 2 diabetes across tertiles of genetic risk defined by hard clustering-derived scores

DQI	PRS/pPRS	Risk Subgroup	HR (95% CI)	p-value	p-interaction*
**AHEI-2010**	Beta-cell + Proinsulin	Low	0.64 (0.38-1.06)	0.082	0.997
		Medium	0.82 (0.54-1.22)	0.327	
		High	0.82 (0.59-1.15)	0.249	
	Beta-cell - Proinsulin	Low	0.88 (0.57-1.36)	0.574	0.976
		Medium	0.72 (0.47-1.11)	0.138	
		High	0.59 (0.41-0.85)	0.005	
	Body fat	Low	0.87 (0.57-1.34)	0.536	0.9
		Medium	0.86 (0.57-1.30)	0.466	
		High	0.68 (0.48-0.96)	0.031	
	Global PRS	Low	0.76 (0.42-1.38)	0.367	0.976
		Medium	0.69 (0.45-1.06)	0.087	
		High	0.82 (0.60-1.12)	0.202	
	Lipodystrophy	Low	0.66 (0.44-1.00)	0.052	0.997
		Medium	0.72 (0.49-1.05)	0.087	
		High	0.86 (0.59-1.25)	0.436	
	Liver and lipid metabolism	Low	0.73 (0.53-0.99)	0.046	0.997
		Medium	0.72 (0.47-1.09)	0.120	
		High	1.03 (0.61-1.74)	0.911	
	Metabolic syndrome	Low	0.78 (0.49-1.24)	0.297	0.9
		Medium	0.80 (0.54-1.18)	0.252	
		High	0.69 (0.48-0.98)	0.040	
	Obesity	Low	0.54 (0.34-0.85)	0.008	0.06
		Medium	0.72 (0.49-1.06)	0.092	
		High	1.18 (0.82-1.70)	0.362	
	Residual glycemic	Low	0.88 (0.57-1.35)	0.557	0.727
		Medium	0.90 (0.61-1.33)	0.595	
		High	0.64 (0.44-0.92)	0.018	
**DASH**	Beta-cell + Proinsulin	Low	0.73 (0.46-1.15)	0.173	0.882
		Medium	1.26 (0.81-1.93)	0.302	
		High	0.75 (0.53-1.08)	0.120	
	Beta-cell - Proinsulin	Low	0.62 (0.40-0.96)	0.032	0.982
		Medium	1.20 (0.80-1.80)	0.385	
		High	0.98 (0.67-1.42)	0.896	
	Body fat	Low	0.84 (0.54-1.30)	0.428	0.626
		Medium	1.20 (0.78-1.83)	0.412	
		High	0.69 (0.48-1.01)	0.055	
	Global PRS	Low	0.66 (0.37-1.18)	0.160	0.599
		Medium	0.88 (0.57-1.35)	0.552	
		High	0.98 (0.70-1.36)	0.888	
	Lipodystrophy	Low	0.77 (0.50-1.18)	0.230	0.882
		Medium	0.79 (0.52-1.18)	0.251	
		High	1.15 (0.79-1.69)	0.468	
	Liver and lipid metabolism	Low	1.00 (0.73-1.37)	0.997	0.753
		Medium	0.66 (0.42-1.03)	0.064	
		High	0.96 (0.54-1.70)	0.897	
	Metabolic syndrome	Low	0.93 (0.58-1.47)	0.745	0.355

		Medium	1.13 (0.73-1.75)	0.573	
		High	0.80 (0.56-1.14)	0.212	
	Obesity	Low	0.75 (0.49-1.15)	0.188	0.467
		Medium	0.85 (0.57-1.28)	0.438	
		High	1.19 (0.81-1.75)	0.367	
	Residual glycemic	Low	0.86 (0.55-1.34)	0.495	0.467
		Medium	1.09 (0.72-1.64)	0.692	
		High	0.75 (0.53-1.08)	0.125	
**DII**	Beta-cell + Proinsulin	Low	1.81 (1.16-2.83)	0.009	0.476
		Medium	1.01 (0.67-1.52)	0.962	
		High	1.01 (0.71-1.44)	0.953	
	Beta-cell - Proinsulin	Low	1.68 (1.09-2.60)	0.020	0.643
		Medium	0.71 (0.48-1.05)	0.090	
		High	1.19 (0.83-1.70)	0.349	
	Body fat	Low	0.92 (0.60-1.42)	0.708	0.476
		Medium	0.78 (0.53-1.15)	0.213	
		High	1.40 (0.96-2.03)	0.082	
	Global PRS	Low	0.89 (0.54-1.47)	0.649	0.643
		Medium	1.04 (0.68-1.61)	0.847	
		High	1.05 (0.76-1.46)	0.768	
	Lipodystrophy	Low	0.79 (0.54-1.17)	0.248	0.643
		Medium	1.34 (0.89-2.03)	0.160	
		High	1.15 (0.79-1.68)	0.456	
	Liver and lipid metabolism	Low	1.12 (0.82-1.52)	0.491	0.476
		Medium	0.95 (0.63-1.43)	0.802	
		High	1.08 (0.61-1.89)	0.797	
	Metabolic syndrome	Low	0.72 (0.47-1.12)	0.143	0.476
		Medium	1.07 (0.73-1.58)	0.736	
		High	1.45 (1.00-2.10)	0.053	
	Obesity	Low	1.33 (0.86-2.07)	0.199	0.896
		Medium	0.76 (0.52-1.11)	0.155	
		High	1.19 (0.83-1.69)	0.338	
	Residual glycemic	Low	1.11 (0.73-1.70)	0.616	0.643
		Medium	0.71 (0.48-1.05)	0.088	
		High	1.54 (1.06-2.24)	0.023	
**MedPyr**	Beta-cell + Proinsulin	Low	0.99 (0.63-1.55)	0.958	0.968
		Medium	1.02 (0.68-1.53)	0.933	
		High	1.11 (0.79-1.55)	0.559	
	Beta-cell - Proinsulin	Low	0.69 (0.44-1.09)	0.112	0.975
		Medium	1.52 (1.04-2.21)	0.031	
		High	0.96 (0.68-1.36)	0.806	
	Body fat	Low	0.90 (0.59-1.38)	0.636	0.968
		Medium	1.05 (0.71-1.54)	0.805	
		High	1.10 (0.77-1.57)	0.602	
	Global PRS	Low	1.01 (0.59-1.72)	0.974	0.968
		Medium	0.81 (0.53-1.24)	0.328	
		High	1.14 (0.82-1.57)	0.441	
	Lipodystrophy	Low	1.27 (0.84-1.92)	0.262	0.968
		Medium	1.03 (0.71-1.51)	0.858	

		High	1.04 (0.72-1.50)	0.839	
	Liver and lipid metabolism	Low	1.01 (0.74-1.37)	0.951	0.968
		Medium	1.30 (0.87-1.94)	0.205	
		High	0.82 (0.49-1.35)	0.424	
	Metabolic syndrome	Low	1.08 (0.71-1.66)	0.715	0.968
		Medium	1.06 (0.71-1.57)	0.785	
		High	0.95 (0.66-1.37)	0.800	
	Obesity	Low	0.84 (0.54-1.32)	0.451	0.968
		Medium	1.18 (0.81-1.72)	0.388	
		High	1.14 (0.80-1.61)	0.477	
	Residual glycemic	Low	0.94 (0.62-1.42)	0.778	0.968
		Medium	0.93 (0.63-1.37)	0.707	
		High	1.13 (0.78-1.62)	0.517	

Model adjusted for age (as underlying time variable), sex, education, physical activity, smoking status, energy intake (except DII), alcohol intake (DASH only).

AHEI: Alternative Healthy Eating Index. DASH: Dietary Approaches to Stop Hypertension. DII: Dietary Inflammatory Index. MedPyr: Mediterranean Diet Pyramid.

*p-interaction (continuous DQI*continuous PRS or pPRS) FDR-adjusted in the case of AHEI-2010, DASH and MedPyr. For DII, p-values were derived from likelihood ratio tests comparing models with and without the interaction term (DII categorical as tertile x continuous PRS), and p-values were FDR adjusted.

S. Table 10: P-interaction for DQIxPRS after excluding baseline CVD cases

DQI	PRS/pPRS	p-interaction
**AHEI-2010**	Smith ALP negative	0.924
	Smith ASAT positive	0.924
	Smith Beta Cell 1	0.793
	Smith Beta Cell 2	0.924
	Smith Bilirubin	0.956
	Smith Cholesterol	0.924
	Smith Global PRS	0.924
	Smith Hyper Insulin	0.924
	Smith Lipodystrophy 1	0.765
	Smith Lipodystrophy 2	0.793
	Smith Liver-Lipid	0.765
	Smith Obesity	0.765
	Smith Proinsulin	0.765
	Smith SHBG-LpA	0.924
	Smith VAT negative	0.924
	Suzuki Beta-cell + Proinsulin	0.924
	Suzuki Beta-cell - Proinsulin	0.924
	Suzuki Body fat	0.765
	Suzuki Global PRS	0.924
	Suzuki Lipodystrophy	0.924
	Suzuki Liver and lipid metabolism	0.956
	Suzuki Metabolic syndrome	0.924
	Suzuki Obesity	0.16
	Suzuki Residual glycemic	0.765
**DASH**	Smith ALP negative	0.538
	Smith ASAT positive	0.807
	Smith Beta Cell 1	0.819
	Smith Beta Cell 2	0.874
	Smith Bilirubin	0.411
	Smith Cholesterol	0.807
	Smith Global PRS	0.807
	Smith Hyper Insulin	0.411
	Smith Lipodystrophy 1	0.819
	Smith Lipodystrophy 2	0.207
	Smith Liver-Lipid	0.538
	Smith Obesity	0.912
	Smith Proinsulin	0.624
	Smith SHBG-LpA	0.807
	Smith VAT negative	0.912
	Suzuki Beta-cell + Proinsulin	0.874
	Suzuki Beta-cell - Proinsulin	0.954
	Suzuki Body fat	0.807
	Suzuki Global PRS	0.819
	Suzuki Lipodystrophy	0.912

	Suzuki Liver and lipid metabolism	0.807
	Suzuki Metabolic syndrome	0.411
	Suzuki Obesity	0.538
	Suzuki Residual glycemic	0.807
**MedPyr**	Smith ALP negative	0.996
	Smith ASAT positive	0.996
	Smith Beta Cell 1	0.996
	Smith Beta Cell 2	0.996
	Smith Bilirubin	0.996
	Smith Cholesterol	0.996
	Smith Global PRS	0.996
	Smith Hyper Insulin	0.996
	Smith Lipodystrophy 1	0.996
	Smith Lipodystrophy 2	0.996
	Smith Liver-Lipid	0.996
	Smith Obesity	0.996
	Smith Proinsulin	0.996
	Smith SHBG-LpA	0.996
	Smith VAT negative	0.996
	Suzuki Beta-cell + Proinsulin	0.996
	Suzuki Beta-cell - Proinsulin	0.996
	Suzuki Body fat	0.996
	Suzuki Global PRS	0.996
	Suzuki Lipodystrophy	0.996
	Suzuki Liver and lipid metabolism	0.996
	Suzuki Metabolic syndrome	0.996
	Suzuki Obesity	0.996
	Suzuki Residual glycemic	0.996
**DII**	Smith ALP negative	0.473
	Smith ASAT positive	0.659
	Smith Beta Cell 1	0.951
	Smith Beta Cell 2	0.519
	Smith Bilirubin	0.447
	Smith Cholesterol	0.447
	Smith Global PRS	0.473
	Smith Hyper Insulin	0.51
	Smith Lipodystrophy 1	0.659
	Smith Lipodystrophy 2	0.51
	Smith Liver-Lipid	0.473
	Smith Obesity	0.473
	Smith Proinsulin	0.931
	Smith SHBG-LpA	0.951
	Smith VAT negative	0.51
	Suzuki Beta-cell + Proinsulin	0.51
	Suzuki Beta-cell - Proinsulin	0.51
	Suzuki Body fat	0.447
	Suzuki Global PRS	0.473
	Suzuki Lipodystrophy	0.659
	Suzuki Liver and lipid metabolism	0.473

	Suzuki Metabolic syndrome	0.447
	Suzuki Obesity	0.718
	Suzuki Residual glycemic	0.51

Model adjusted for age (as underlying time variable), sex, education, physical activity, smoking status, energy intake (except DII), alcohol intake (DASH only).
AHEI: Alternative Healthy Eating Index. DASH: Dietary Approaches to Stop Hypertension. DII: Dietary Inflammatory Index. MedPyr: Mediterranean Diet Pyramid.
*p-interaction (continuous DQI*continuous PRS or pPRS) FDR-adjusted in the case of AHEI-2010, DASH and MedPyr. For DII, p-values were derived from likelihood ratio tests comparing models with and without the interaction term (DII categorical as tertile x continuous PRS), and p-values were FDR adjusted.
